# Accelerating cartilage regeneration with DNA-SF hydrogel sustained release system-based cartilage organoids

**DOI:** 10.1186/s40779-025-00625-z

**Published:** 2025-07-28

**Authors:** Cong-Yi Shen, Qi-Rong Zhou, Xiang Wu, Xin-Yu Han, Qin Zhang, Xiao Chen, Yu-Xiao Lai, Long Bai, Ying-Ying Jing, Jian-Hua Wang, Cheng-Long Wang, Zhen Geng, Jia-Can Su

**Affiliations:** 1 Institute of Translational Medicine, Shanghai University, Shanghai, 200444 China; 2 MedEng-X Institutes, Shanghai University, Shanghai, 200444 China; 3 Organoid Research Center, Shanghai University, Shanghai, 200444 China; 4 School of Medicine, Shanghai University, Shanghai, 200444 China; 5 National Center for Translational Medicine (Shanghai) SHU Branch, Shanghai University, Shanghai, 200444 China; 6 Department of Orthopedics, Xinhua Hospital, Shanghai Jiao Tong University School of Medicine, Shanghai, 200092 China; 7 Centre for Translational Medicine Research and Development, Institute of Biomedical and Health Engineering, Shenzhen Institute of Advanced Technology, Chinese Academy of Sciences, Shenzhen, 518055 Guangdong China

**Keywords:** Cartilage organoid (COs), Glucosamine, TD-198946, DNA-silk fibroin hydrogel, Chondrogenesis, Cartilage repair

## Abstract

**Background:**

Cartilage repair remains a considerable challenge in regenerative medicine. Despite extensive research on biomaterials for cartilage repair in recent years, issues such as prolonged repair cycles and suboptimal outcomes persist. Organoids, miniature three-dimensional (3D) tissue structures derived from the directed differentiation of stem or progenitor cells, mimic the structure and function of natural organs. Therefore, the construction of cartilage organoids (COs) holds great promise as a novel strategy for cartilage repair.

**Methods:**

This study employed a digital light processing system to perform 3D bioprinting of a DNA-silk fibroin (DNA-SF) hydrogel sustained-release system (DSRGT) with bone-marrow mesenchymal stem cells (BMSCs) to construct millimeter-scale cerebral organoids. COs at different developmental stages were characterized, and the COs with the best cartilage phenotype were selected for in vivo cartilage repair in a rat articular cartilage defect model.

**Results:**

This study developed a DSRGT by covalently grafting glucosamine (which promotes cartilage matrix synthesis) and TD-198946 (which promotes chondrogenic differentiation) onto a hydrogel using acrylic acid-polyethylene glycol-N-hydroxysuccinimide (AC-PEG-NHS). In vitro, 4-week COs exhibited higher SRY-box transcription factor 9 (SOX9), type II collagen (Col II), and aggrecan (ACAN) expression and lower type I collagen (Col I) and type X collagen (Col X) expression, indicating that 4 weeks is the optimal culture duration for hyaline cartilage development. In vivo, the mitogen-activated protein kinase (MAPK) signaling pathway was upregulated in 4-week COs, enabling cartilage repair within 8 weeks. Transcriptomic analysis revealed that cartilage regenerated with 4-week COs presented gene expression profiles resembling those of healthy cartilage.

**Conclusions:**

This study employs DSRGT to construct COs, providing an innovative strategy for the regeneration of cartilage defects.

**Supplementary Information:**

The online version contains supplementary material available at 10.1186/s40779-025-00625-z.

## Background

Osteoarthritis (OA) is a common degenerative joint disease characterized by articular cartilage degradation and represents one of the leading causes of adult disability worldwide, affecting over 595 million people and posing significant economic burdens on both patients and society [1–4]. Current cartilage repair strategies, including microfracture, autologous chondrocyte transplantation, and allogeneic cartilage transplantation, are widely used in clinical practice but have various limitations [5–9]. Therefore, novel technologies and approaches are urgently needed to address the challenges associated with cartilage repair.

In recent years, researchers have extensively explored the use of biomaterials for cartilage defect repair [10]. However, implantation of biomaterials for repairing cartilage defects typically requires endogenous cell recruitment, proliferation, and matrix secretion, leading to prolonged repair periods [11, 12]. Organoids, which are derived from the directed differentiation of stem or progenitor cells, exhibit structural and functional characteristics of native organs [13–15]. They also demonstrated self-renewal and self-organization capabilities. Compared with biomaterials, cartilage organoids (COs) do not rely on endogenous cell recruitment and proliferation, potentially expediting the cartilage repair process. Recently, Abe et al. [16] constructed COs in vitro using induced pluripotent stem cells from a cynomolgus monkey. When COs was transplanted into the knee cartilage defect model of cynomolgus monkeys, it was found to successfully survive, integrate, and remodel into articular cartilage in vivo. Wen et al. [17] used human polydactyly chondrocytes and serum-free customized medium for three-dimensional (3D) culture to generate macro-COs. These macro-COs significantly promoted osteochondral defect healing. Thus, constructing COs as grafts for cartilage regeneration seems to be an ideal strategy for accelerating cartilage repair.

Currently, the most common methods for constructing COs include scaffold-free self-organization and coculture with biomaterials [18, 19]. In contrast to scaffold-free self-organization, co-culture with biomaterials provides 3D networks similar to the extracellular matrix (ECM) of cartilage [20]. This network supported chondrocyte proliferation and maintained physiological function. When organoids are co-cultured with biomaterials, the structure and size can be regulated through biomaterial design. In a previous study, a DNA-silk fibroin (DNA-SF) was successfully developed to promote the chondrogenic differentiation of bone-marrow mesenchymal stem cells (BMSCs) by regulating surface stiffness [21]. Furthermore, microspheres prepared from DNA-SF hydrogel and modified with Arg-Gly-Asp (RGD) peptides were used as scaffolds for constructing COs [22]. However, 14 d of chondrogenic induction did not support complete cartilage ECM synthesis, leading to the formation of immature CO precursors. Additionally, Chen et al. [23] demonstrated that prolonged in vitro culture led to the dedifferentiation of chondrocytes. This process resulted in a fibroblast-like morphology with abnormal ECM deposition, which eventually progressed to a pathological hypertrophic state.

Glucosamine (Glu) can stimulate BMSCs to produce an ECM resembling native cartilage and maintain the stability of chondrocytes and the cartilage ECM [24, 25]. TD-198946, a thiophene-benzimidazole derivative, is a highly effective chondrogenic agent [26, 27]. Unlike other chondrogenic agents [e.g., bone morphogenetic protein 2 (BMP2) and transforming growth factor-β3 (TGF-β3)], TD-198946 not only promoted the differentiation of BMSCs into hyaline cartilage but also inhibited dedifferentiation and hypertrophy. However, simple drug encapsulation in hydrogels often leads to burst release. Acrylic acid-polyethylene glycol-N-hydroxysuccinimide (AC-PEG-NHS) is a bifunctional ester-based covalent crosslinker [28]. Its NHS ester group reacts with the amino groups on Glu and TD-198946, whereas its acrylate group reacts with silk fibroin methacrylate (SilMA). Thus, AC-PEG-NHS enables the covalent grafting of Glu and TD-198946 onto the DNA-SF hydrogel.

Overall, this study employed AC-PEG-NHS to covalently anchor Glu and TD-198946 onto the DNA-SF hydrogel network. The purpose of this modification is to enhance the functionality of the DNA-SF hydrogel so that it can support sustained chondrogenic differentiation of BMSCs, promote ECM synthesis, and prevent dedifferentiation and hypertrophy during long-term culture. These features are expected to promote the formation of long-term cultured and mature COs. By integrating digital light processing (DLP) 3D bioprinting technology, millimeter-scale COs are fabricated. This work aims to investigate the mechanism of action of COs in cartilage defect repair, and to analyze the differences between regenerated cartilage from COs after repair and healthy cartilage. This research provides significant insights for the development of biomaterials for the COs construction and cartilage defect regeneration.

## Materials and methods

### Preparation of DNA-silk fibroin hydrogel sustained-release system (DSRGT)

To prepare the DSRGT solution, the following components were dissolved and mixed in PBS (pH 7.4) at 37 °C: 10 wt% SilMA (EFL, China), 0.25 wt% lithium phenyl-2,4,6-trimethylbenzoylphosphinate (LAP), 5 wt% RGD-containing peptide modified with D-Phe-Lys and an AC functional group (Pep-RGDfKAC), 100 nmol/L TD-198946 (MCE, USA), 10 mmol/L Glu (MCE, USA), 4.5 wt% AC-PEG-NHS (EFL, China), and 500,000 nmol/L ssDNA (Sangon Biotech, China). TD-198946 was pre-dissolved in dimethyl sulfoxide (DMSO) before addition. The DSRGT solution underwent gelation under 365 nm ultraviolet (UV) light and was subsequently used for experiments. 3D printing was performed using a DLP printer (EFL-BP-8601 Pro, EFL, China), with the following parameters: light intensity of 20 mW/cm^2^, exposure time of 17 s, and layer height of 100 μm. The single-stranded DNA (ssDNA) sequences used to construct this network are detailed in Additional file 1: Table S1.

### Cell culture

Rat BMSCs were obtained from a cell bank (Shanghai, China). The cells were cultured in α-minimum essential medium (α-MEM, Gibco, USA) supplemented with 10% fetal bovine serum (Gibco, USA) and 1% penicillin–streptomycin (Gibco, USA) at 37 °C in a 5% CO₂ atmosphere. The culture medium was replaced every 2 days, and third-passage BMSCs were used for experiments.

### Cell viability and cytotoxicity staining

Four hydrogel precursor solutions, designated as DSR, DSRG, DSRT, and DSRGT, were filtered through 0.22 μm membranes (200 μl/well) and photopolymerized in 24-well plates (Nest, China), forming the corresponding experimental groups. Subsequently, 1 × 10^4^ cells were seeded into each well. Mixing the hydrogel precursors with cells (cell density 1 × 10⁶ cells/ml), and cell-laden spheres were fabricated using a DLP system, maintaining the same group designations (DSR, DSRG, DSRT, and DSRGT). After culturing for 1, 3, and 5 d, cells were stained with the calcein AM/PI double staining kit (Beyotime, China) and incubated for 30 min. Cell viability and death were then observed using an inverted fluorescence microscope (Olympus CKX3-SLP, Japan).

### Chondrogenic induction

Four hydrogel precursor solutions (DSR, DSRG, DSRT, and DSRGT; 200 μl per well) were added to a 24-well plate (Nest, China) and photopolymerized. A total of 1 × 10^4^ BMSCs were seeded into each well and cultured in chondrogenic induction medium. The chondrogenic induction medium consisted of 1 mmol/L sodium pyruvate (Sigma, USA), 10 ng/ml TGF-β3 (Meilunbio, China), 0.1 μmol/L dexamethasone (Sigma, USA), 1% insulin-transferrin-selenium (ITS) premix (Oricellbio, China), and 1 μmol/L ascorbate-2-phosphate (Sigma, USA). The medium was changed every 2 days.

### CO culture and evaluation

Mixing the DSRGT precursor with BMSCs (cell density: 1 × 10⁶ cells/ml), and cell-laden spheres were fabricated using a DLP printer. The DSRGT cell-laden spheres were cultured in chondrogenic induction medium for up to 6 weeks, with medium refreshed every 2 days. COs cultured for 1, 2, 4, and 6 weeks were collected and fixed in 4% paraformaldehyde for subsequent characterization.

### Source of animals and study design

In this study, 6-week-old male SD rats (approximately 150 g, *n* = 48) were purchased from Changzhou Kevins Experimental Animal Co., Ltd. The rats were randomly assigned to cages by an animal facilities technician. Prior to any procedures, the cages were randomly selected and assigned to each group by an individual not involved in the study. All in vivo experiments, as well as the investigators responsible for data collection and analysis, were blinded. Animal care was provided in accordance with the “Guide for the Care and Use of Laboratory Animals” in China. This study was approved by the Animal Ethics Committee of Shanghai University (YS 2024–171), and every effort was made to minimize animal suffering.

The animals were randomly divided into 4 groups: sham (positive control), control (untreated defects covered with fibrin glue), DSRGT (defects treated with DSRGT and fibrin glue), and CO (defects treated with 4-week cultured COs and fibrin glue). Under general anesthesia (80 mg/kg sodium pentobarbital), a cartilage defect model with a diameter of 2 mm and depth of 0.2 mm was created in the central area of the distal femoral condyle. DSRGT or COs (cultured for 4 weeks in vitro) were then transplanted into the defect site and fixed with fibrin glue. After the surgery, the knee joint capsule and skin were sutured and disinfected, and standardized postoperative care was provided. At week 4 post-surgery, the rats were euthanized, and their femora were collected for initial analysis. At week 8 post-surgery, femora, heart, liver, spleen, lungs, kidneys, and abdominal aorta blood samples were collected for further analysis.

### Transcriptomics and data processing

Cell samples were directly extracted using Trizol reagent (Beyotime, China). Tissue samples were frozen in liquid nitrogen immediately after collection, ground into powder, and then extracted using Trizol reagent (Beyotime, China). The corresponding RNA libraries were constructed by OE Biotech Co., Ltd., China, and transcript products were analyzed through high-throughput sequencing. Sequencing data were processed using DESeq software to identify differentially expressed genes (DEGs), with significant genes selected based on a *P*-value < 0.05 and a |log_2_ fold change|> 1. Furthermore, principal component analysis (PCA), enrichment analysis of Kyoto Encyclopedia of Genes and Genomes (KEGG) pathways, Gene Ontology (GO), and short time-series expression miner (STEM) were conducted employing advanced algorithms within the R package.

Other detailed methods for material synthesis and characterization, as well as in vitro and in vivo experiments, are provided in the Additional file 1: Materials and methods.

### Statistical analysis

All experimental data were obtained from a minimum of 3 independent trials and are presented as mean ± standard deviation (SD). For pairwise comparisons between two groups, unpaired two-tailed Student’s *t*-tests were applied. When comparing 3 or more groups, One-way analysis of variance (ANOVA) was employed, followed by Tukey’s post hoc test. Nonparametric tests were used for comparisons involving non-normal distributions. Image color intensity quantification was conducted using ImageJ software. Statistical analysis was performed using Prism Version 9 and SPSS 22.0. *P* < 0.05 was considered statistically significant.

## Results

### Synthesis and characterization of DSRGT

**Fig. 1:**
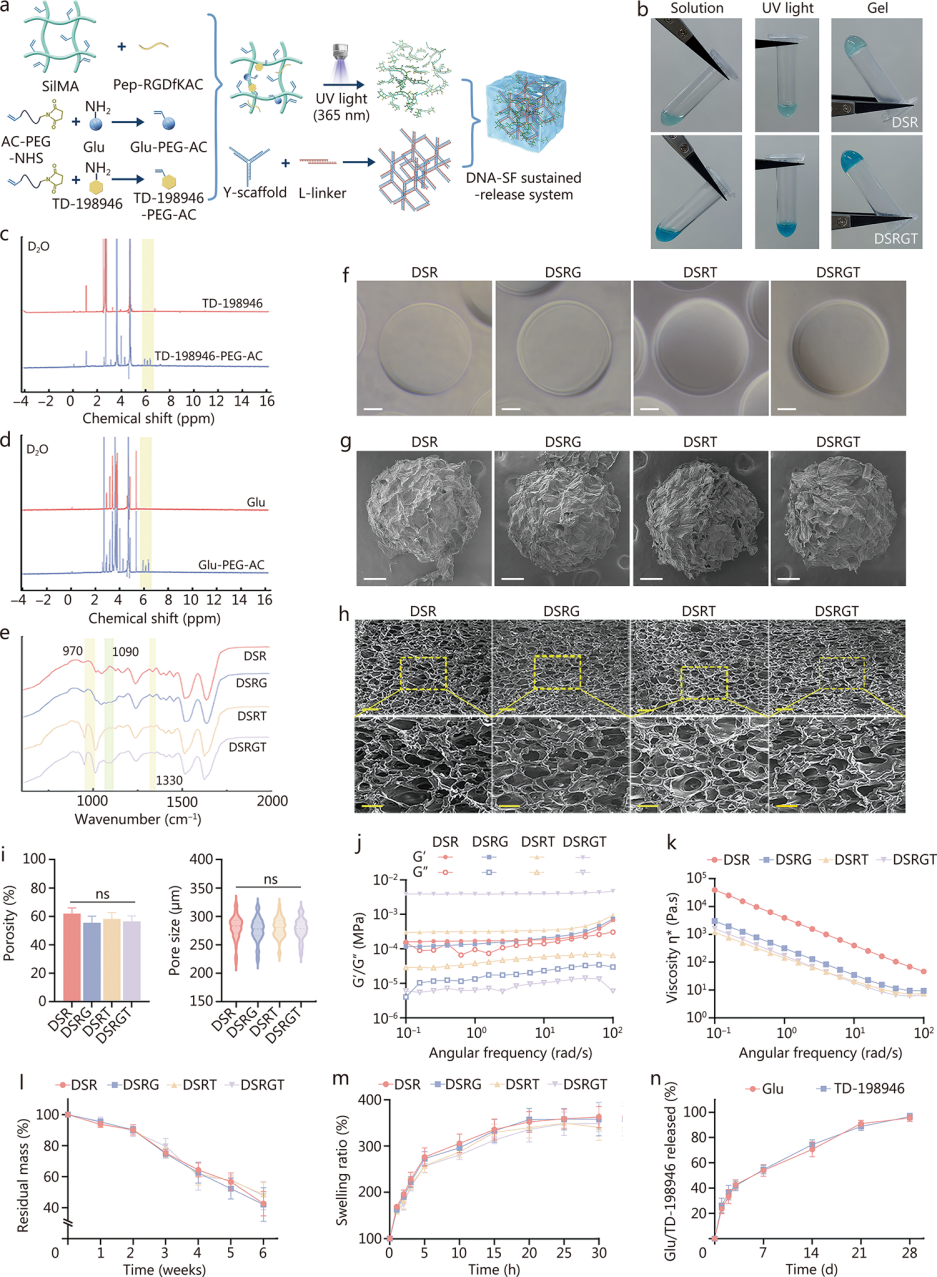
Synthesis and characterization of DSRGT. **a** Schematic representation of the synthesis and molecular interaction mechanism of DSRGT. **b** Solution-to-gel transition. **c** ^1^H nuclear magnetic resonance (NMR) spectra of TD-198946 and TD-198946-PEG-AC in D_2_O. **d** ^1^H NMR spectra of Glu and Glu-PEG-AC in D_2_O. **e** Fourier transform infrared spectroscopy (FTIR) spectra of DSR, DSRG, DSRT, and DSRGT. **f** Macroscopic images of spheres printed with DSR, DSRG, DSRT, and DSRGT in the DLP system. Scale bar = 500 µm. **g** Field-emission scanning electron microscopy (FE-SEM) images of spheres printed with DSR, DSRG, DSRT, and DSRGT in the DLP system. Scale bar = 500 µm. **h** FE-SEM images of bulk hydrogels: DSR, DSRG, DSRT, and DSRGT. **i** Porosity (*n* = 6) and pore sizes (*n* = 150) of DSR, DSRG, DSRT, and DSRGT. Scale bar = 400 µm (upper) and 200 µm (lower). **j** Rheological curves of DSR, DSRG, DSRT, and DSRGT. **k** Viscosity curves of DSR, DSRG, DSRT, and DSRGT. **l** Degradation rates of DSR, DSRG, DSRT, and DSRGT (*n* = 6). **m** Swelling ratios of DSR, DSRG, DSRT, and DSRGT (*n* = 6). **n** Release profiles of Glu and TD-198946 from DSRGT. Data are presented as mean ± SD. One-way ANOVA and Tukey’s multiple-comparisons test were used for data analysis. ns non-significant, SilMA silk fibroin methacrylate, Glu glucosamine, AC-PEG-NHS acrylic acid-polyethylene glycol-N-hydroxysuccinimide, DNA-SF DNA-silk fibroin, Pep-RGDfKAC RGD-containing peptide modified with D-Phe-Lys and an AC functional group, UV ultraviolet, DSR RGD-modified DNA-silk fibroin hydrogel, DSRG Glu-containing DSR, DSRT TD-198946-containing DSR, DSRGT DNA-silk fibroin hydrogel sustained-release system, DLP digital light processing, G' storage modulus, G'' loss modulus, η* complex viscosity

In this study, DSRGT was composed of 2 networks. The first network consisted of an RGD-modified DNA-silk fibroin hydrogel (DSR), which was covalently grafted with Glu and TD-198946. The second network was a DNA supramolecular network based on Y-scaffold ssDNA and L-Linker ssDNA (Fig. 1a). To verify that the DNA supramolecular hydrogel can be effectively assembled via base-pairing interactions, agarose gel electrophoresis was performed (Additional file 1: Fig. S1a). As shown, the ssDNA sequences were successfully assembled into Y-scaffolds and L-linkers. Furthermore, the Y-scaffolds and L-linkers were able to form single units for constructing the DNA supramolecular network through complementary sticky-end hybridization. After exposure to 365 nm UV light for 30 s, both DSR and DSRGT successfully formed hydrogels, indicating that the addition of Glu-PEG-AC and TD-198946-PEG-AC did not affect the gelation ability of the hydrogel (Fig. 1b). In the ^1^H nuclear magnetic resonance (NMR) spectra, TD-198946-PEG-AC and Glu-PEG-AC exhibited characteristic double-bond peaks near 6 ppm (highlighted by yellow boxes), confirming the successful double-bond modification of Glu and TD-198946 by AC-PEG-NHS (Fig. 1c, d; Additional file 1: Fig. S1b). Subsequently, Fourier transform infrared spectroscopy (FTIR) was used to analyze DSR, Glu-containing DSR (DSRG), TD-198946-containing DSR (DSRT), and DSRGT (Fig. 1e). The FTIR spectra of DSRG and DSRGT showed a significant reduction in the absorption peak at 1090 cm⁻^1^. This change was attributed to interactions between the abundant hydroxyl groups in Glu and the C-O groups in SilMA, leading to altered vibration modes of the C-O bonds. TD-198946, which contains aromatic and heterocyclic rings, exhibited distinct FTIR absorption peaks at 970 cm⁻^1^ and 1330 cm⁻^1^ in the spectra of DSRT and DSRGT, corresponding to C–H bending of aromatic rings and C–N vibrations of heterocycles, respectively, confirming its successful incorporation. Fourier self-deconvolution was applied to the infrared absorption spectra in the range of 1600–1700 cm⁻^1^ to calculate the peak area ratio of β-sheets (Additional file 1: Fig. S1c, d). The results showed that although Glu grafting led to a reduction in β-sheet content [(36.02 ± 2.53)%], the β-sheet content in the group co-grafted with Glu and TD-198946 [(48.05 ± 2.51)%] was not significantly different from that of the DSR group [(47.82 ± 1.64)%]. These findings indicate that the co-grafting of Glu and TD-198946 does not affect the β-sheet content, which is closely associated with surface rigidity.

A 3D spherical model with a diameter of 2.5 mm was subsequently created in 3D Max software for printing (Additional file 1: Fig. S1e). Traditional 3D bioprinting typically involves layer-by-layer printing, whereas the DLP system utilizes projection-based photopolymerization. This approach enables simultaneous solidification across multiple areas, allowing the printing of multiple samples in a single process. With the DLP system, the precursor solutions undergo layer-by-layer photopolymerization to precisely construct the desired 3D spherical structures. Samples printed with the DLP system exhibited highly similar morphologies, even with different precursor solutions, ensuring high consistency and accuracy (Fig. 1f). In addition, key parameters of the DLP system were optimized, including light intensity, exposure time, and layer height. Within a specific range of parameters (light intensity: 15–30 mW/cm^2^; exposure time: 12–25 s; layer height ≤ 150 μm), uniform spherical arrays were consistently produced, demonstrating high reproducibility (Additional file 1: Fig. S2a). Furthermore, DSRGT was used to print other models, such as ear-shaped and dual-layered structures (Additional file 1: Fig. S2b). The ear-shaped model can be used for orthopedic correction of congenital ear deformities, while the dual-layered structures are suitable for constructing dual-layered COs.

Under the DLP system, DSRGT showed high scalability for building COs with different applications. Field-emission scanning electron microscopy (FE-SEM) was used to observe the lyophilized spheres printed by the DLP system. As shown in Fig. 1g, all groups of spheres exhibited porous surface structures. To further evaluate the pore size and porosity, FE-SEM was performed on bulk hydrogels prepared directly from the precursor solutions. As shown in Fig. 1h, all 4 hydrogel groups displayed uniform porous structures. The porosity of the hydrogels was (61.90 ± 4.11)% for DSR, (55.46 ± 4.68)% for DSRG, (58.05 ± 4.59)% for DSRT, and (56.44 ± 3.88)% for DSRGT. The corresponding pore sizes were (280.7 ± 20.59) μm for DSR, (276.1 ± 20.93) μm for DSRG, (279.6 ± 17.32) μm for DSRT, and (277.6 ± 18.75) μm for DSRGT (Fig. 1i). DSR exhibited the highest porosity and pore size, followed by DSRT. The introduction of AC-PEG-NHS increased the degree of crosslinking of the hydrogel, but the impact was minimal, since the content of TD-198946-PEG-AC was much lower than that of Glu-PEG-AC. No significant differences were found in porosity or pore size among the groups, suggesting that the incorporation of Glu-PEG-AC and TD-198946-PEG-AC did not significantly interfere with these processes. The uniform porous structure of the hydrogels provided an excellent microenvironment for cell migration and adhesion.

The rheological properties of the 4 precursor solutions were evaluated. The frequency sweep curves of each group are shown in Fig. 1j. All groups exhibited typical hydrogel mechanical behavior, with the storage modulus (G') consistently higher than the loss modulus (G''), indicating that all the precursor solutions successfully formed relatively stable hydrogels under 365 nm UV light, making them suitable for 3D printing. The viscosity changes of the precursor solutions were also assessed. All the hydrogels exhibited shear-thinning behavior, where the viscosity decreased under shear stress. Notably, the incorporation of Glu-PEG-AC and TD-198946-PEG-AC resulted in a reduction in hydrogel viscosity. This phenomenon was attributed to the low viscosity and high fluidity of the PEG molecules in the covalent crosslinker AC-PEG-NHS, which reduced the overall viscosity of the hydrogels (Fig. 1k). To simulate the degradation behavior of hydrogels during long-term in vitro culture of COs, the degradation of the 4 hydrogel spheres in chondrogenic induction medium was evaluated (Fig. 1l). Over 6 weeks, the residual mass of all the hydrogel spheres gradually decreased, with no significant differences between groups. Specifically, at 2 weeks, the residual mass was (90.11 ± 2.75)%, which decreased to (62.27 ± 6.76)% at 4 weeks and (45.28 ± 8.85)% at 6 weeks. This moderate degradation rate provided favorable conditions for CO development, ensuring that the hydrogels supported cell growth and tissue formation during cell expansion while gradually degrading during long-term culture without impairing cartilage development. The swelling behavior of the 4 hydrogel spheres in chondrogenic induction medium was further monitored (Fig. 1m). The DSR group presented the highest swelling ratio, but no significant differences were observed among the 4 hydrogel spheres. All the hydrogel spheres rapidly swelled to (265.72 ± 17.55)% within 5 h and stabilized at (346.29 ± 24.30)% after 20 h. This outcome indicated that the hydrogel spheres possessed excellent water absorption properties, providing a hydrated environment conducive to CO growth. Finally, DSRGT was immersed in PBS, and the concentrations of released Glu and TD-198946 were measured at predetermined time points (Fig. 1n). The results revealed no significant differences in the release rates of Glu and TD-198946. Both showed a rapid release phase within the first 3 d, reaching (42.26 ± 3.67)%, followed by a steady release phase until day 21, with a cumulative release of (89.85 ± 2.98)%. By day 28, nearly all the nutrients were released, with a final release rate of (95.94 ± 2.59)%. For comparison, Glu and TD-198946 were mixed directly with DSR without covalent grafting, and the mixture was immersed in PBS. The release rates were measured at the same time points. The results revealed no significant differences between Glu and TD-198946 in terms of release rate. However, their release was much faster, with (61.64 ± 37.33)% released on day 1 and nearly complete release by day 3 (Additional file 1: Fig. S2c). These findings demonstrated that covalent grafting significantly prolonged and controlled the release process, ensuring slow and uniform nutrient release over an extended period. This sustained-release characteristic extended the nutrient supply duration, thereby supporting the long-term culture of COs.

### Biocompatibility of DSRGT

**Fig. 2:**
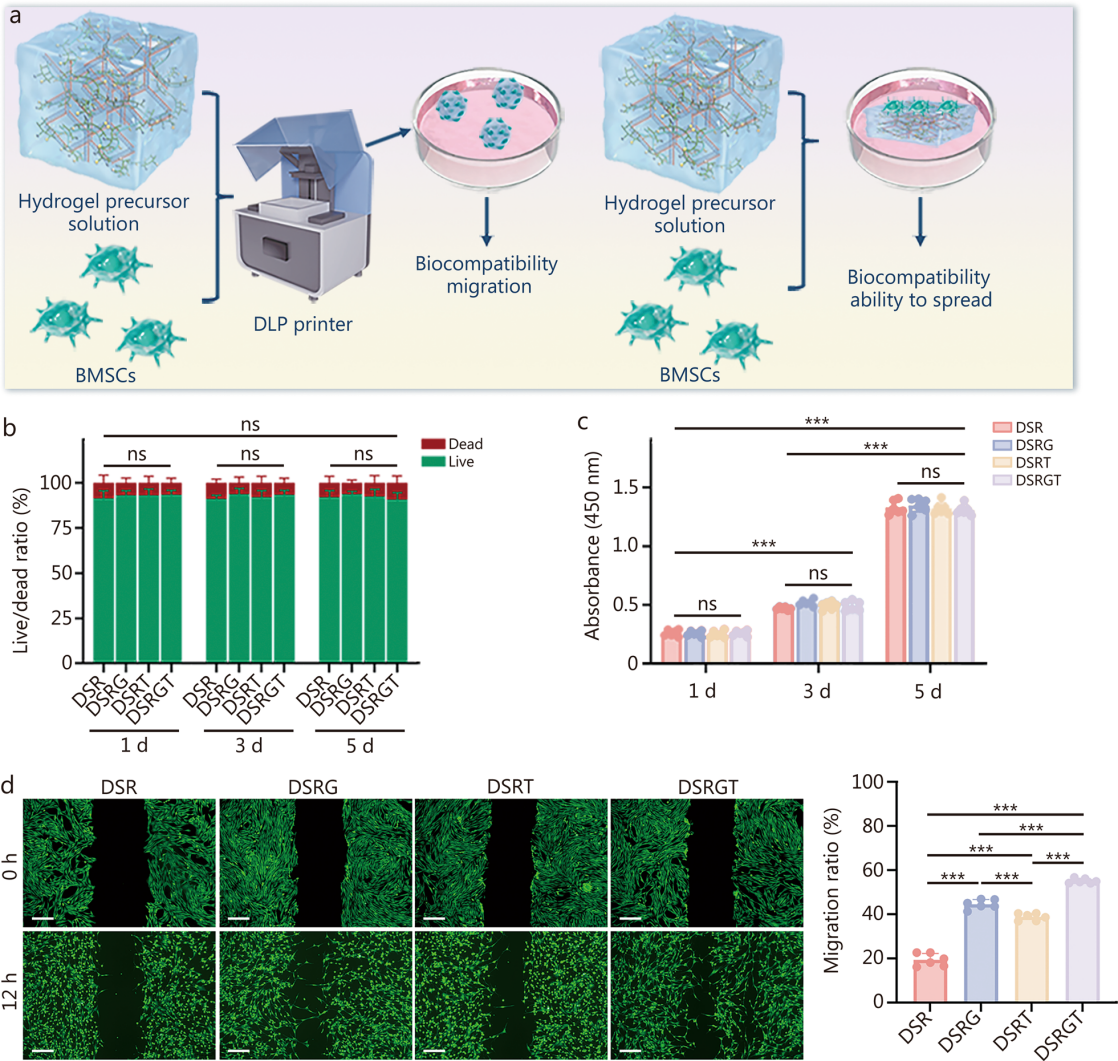
Effects of DSR, DSRG, DSRT, and DSRGT on the biocompatibility and migration of BMSCs. **a** Schematic of the experiment. **b** Live/dead cell ratios of BMSCs cultured in DSR, DSRG, DSRT, and DSRGT for 1, 3, and 5 d in 3D culture (*n* = 3). **c** Cell counting kit-8 (CCK-8) assay results of cells cultured in DSR, DSRG, DSRT, and DSRGT for 1, 3, and 5 d (*n* = 6). **d** Migration behavior and quantitative analysis of BMSCs on DSR, DSRG, DSRT, and DSRGT after 12 h of culture. Scale bar = 200 µm. Data are presented as mean ± SD. One-way ANOVA and Tukey’s multiple-comparisons test were used for data analysis. ***P < 0.001, ns non-significant. DSR RGD-modified DNA-silk fibroin hydrogel, DSRG Glu-containing DSR, DSRT TD-198946-containing DSR, DSRGT DNA-silk fibroin hydrogel sustained-release system, DLP digital light processing, BMSCs bone-marrow mesenchymal stem cells

To evaluate the in vitro biocompatibility of the DSR, DSRG, DSRT, and DSRGT hydrogels, BMSCs were seeded onto each hydrogel for coculture. Additionally, precursor solutions were mixed with BMSCs and printed into cell-laden spheres with the DLP system for 5 d of coculture (Fig. 2a). Live/dead cell staining was performed on days 1, 3, and 5 to assess the viability of the BMSCs on hydrogels and in cell-laden spheres (Additional file 1: Fig. S3a, b). The results revealed high BMSC survival rates (green fluorescence) with minimal dead cells (red fluorescence) across all groups, with no significant differences in live/dead ratios among groups (Fig. 2b). Cell counting kit-8 (CCK-8) assays revealed significant BMSC proliferation in the DSR, DSRG, DSRT, and DSRGT groups from day 1 to day 5, with no significant differences between time points (Fig. 2c). These findings confirmed the excellent biocompatibility of all the hydrogel groups. Glu, a natural glycosaminoglycan (GAG), is known to increase cell adhesion and migration [29]. Cytoskeletal staining of cell-laden spheres after 1 d of culture revealed that the BMSCs in the DSRG and DSRGT groups exhibited uniform spreading throughout the material, whereas those in the DSR and DSRT groups showed less extensive spreading. Compared with the DSR group, the DSRT group demonstrated slightly improved cell spreading (Additional file 1: Fig. S3c). Cell migration plays a critical role in the development of COs. To further investigate the effects of DSR, DSRG, DSRT, and DSRGT on BMSC migration, a scratch assay was performed (Fig. 2d). After 12 h of culture, the DSRGT group significantly increased the migration rate of the BMSCs, reaching (55.09 ± 1.08)%, which was higher than that of all other groups. Additionally, the migration rate in the DSRT group [(38.82 ± 1.60)%] was significantly higher than that in the DSR group. The Transwell assay further demonstrated that the DSRGT group significantly promoted cell migration (Additional file 1: Fig. S4a). Similarly, the number of migrated cells in the DSRT group was also significantly higher compared to the DSR group, reaching 158.0 ± 17.51. This effect was attributed to TD-198946, which activates the Notch3 signaling pathway in stem cells, a pathway known to promote cell migration [30]. Western blotting analysis was performed to verify this observation, confirming that grafted TD-198946 can also activate the Notch3 signaling pathway in stem cells (Additional file 1: Fig. S4b). In conclusion, DSRGT not only demonstrated excellent biocompatibility but also enhanced the spreading and migration of BMSCs in 3D culture. These results highlight the potential of DSRGT as an ideal material for CO construction in 3D culture systems.

### Chondrogenic differentiation capacity of DSRGT

**Fig. 3:**
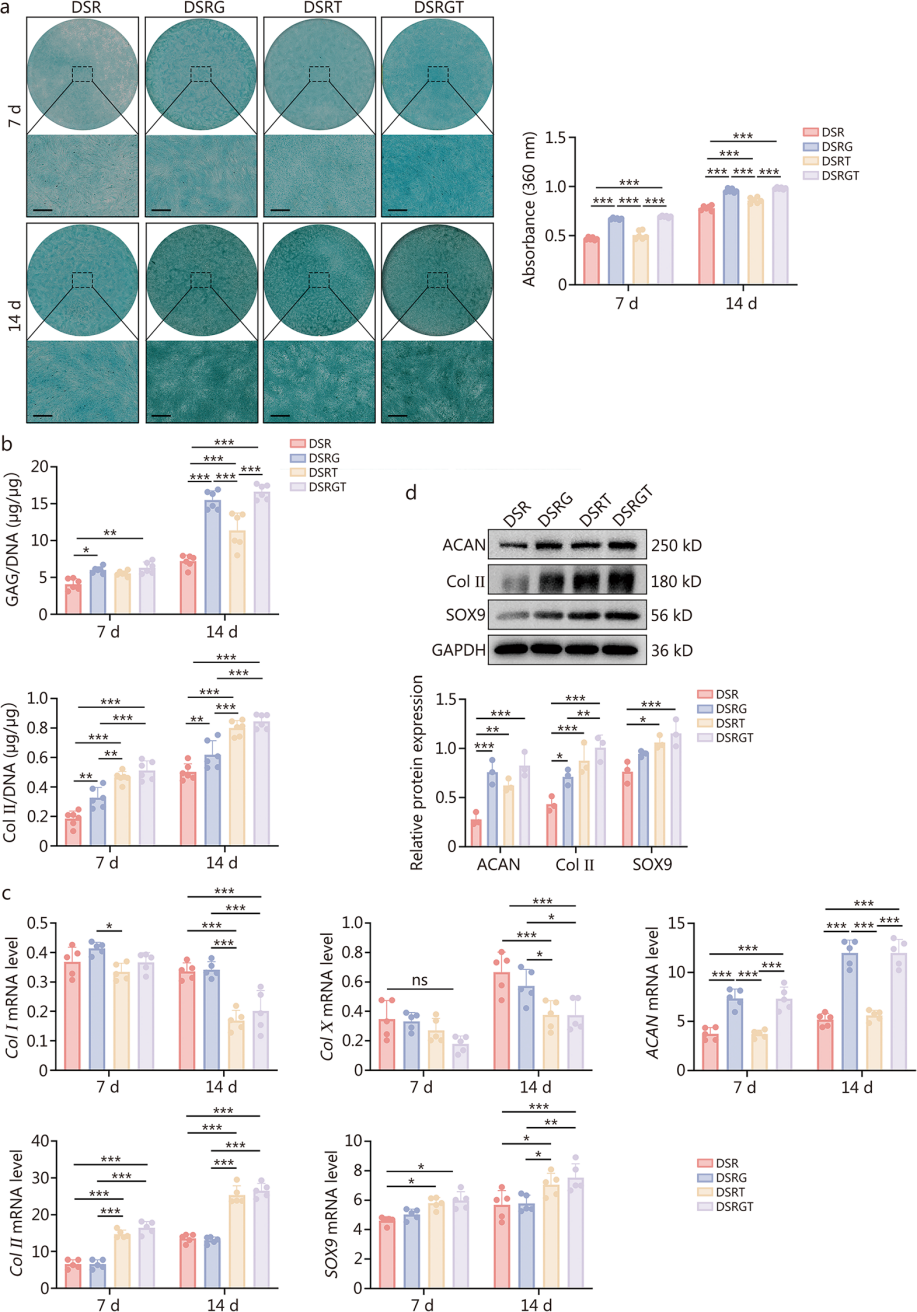
Chondrogenic differentiation capacity of BMSCs by DSR, DSRG, DSRT, and DSRGT. **a** Alcian blue staining and quantitative analysis of BMSCs cocultured with DSR, DSRG, DSRT, and DSRGT on days 7 and 14 (*n* = 6). Scale bar = 200 µm. **b** Quantitative analysis of GAG and Col II contents (*n* = 6). **c** Quantitative real-time polymerase chain reaction (qRT-PCR) results for *Col I*, *Col X, ACAN*, *Col II,* and *SOX9* expression on days 7 and 14 (*n* = 6). **d** Western blotting and quantitative analysis for ACAN, Col II, and SOX9 (*n* = 3). Data are presented as mean ± SD. One-way ANOVA and Tukey’s multiple-comparisons test were used for data analysis. *P < 0.05, **P < 0.01, ***P < 0.001, ns non-significant. DSR RGD-modified DNA-silk fibroin hydrogel, DSRG Glu-containing DSR, DSRT TD-198946-containing DSR, DSRGT DNA-silk fibroin hydrogel sustained-release system, GAG glycosaminoglycan, GAPDH glyceraldehyde-3-phosphate dehydrogenase, Col II type II collagen, ACAN aggrecan, SOX9 SRY-box transcription factor 9, Col X type X collagen, Col I type I collagen, BMSCs bone-marrow mesenchymal stem cells

To investigate the effects of DSR, DSRG, DSRT, and DSRGT hydrogels on the chondrogenic differentiation of BMSCs, a 14-day chondrogenic induction was conducted following prior evaluation of biocompatibility, cell spreading, and migration-promoting abilities. To assess chondrogenic markers, particularly GAG content, Alcian blue staining was performed for qualitative and quantitative analyses. After 7 and 14 d of culture, the staining intensity of the DSRG and DSRGT groups was significantly higher than that of the other groups (Fig. 3a). This difference was especially prominent on day 14, with DSRG and DSRGT exhibiting the strongest staining, followed by DSRT, while DSR showed the weakest staining. These results indicated that GAG synthesis during chondrogenic differentiation was more pronounced in the DSRG and DSRGT groups. Quantitative analysis further confirmed that GAG production in the DSRG and DSRGT groups was significantly higher than that in the other groups. No significant difference was observed between the DSRG and DSRGT groups. This finding was attributed to Glu, which serves as a direct donor for the synthesis of GAG and proteoglycans, effectively promoting GAG secretion by BMSCs.

To further investigate the effects of different hydrogels on the chondrogenic differentiation of BMSCs, the GAG and type II collagen (Col II) contents in the hydrogels were quantitatively assessed using the 1,9-dimethylmethylene blue (DMMB) assay and enzyme-linked immunosorbent assay (ELISA). As shown in Fig. 3b, the GAG content in the DSRG and DSRGT groups was observed to be higher at day 14 than at day 7. These levels were significantly higher than those of the other groups, further validating the results of the staining experiments. Similarly, the Col II levels in the DSRT and DSRGT groups were higher on day 14 than on day 7, and significantly exceeded those of the other groups at the same time point. This increase was attributed to TD-198946, which effectively promoted Col II secretion during the chondrogenic induction of BMSCs. The expression of chondrogenesis-related genes during 7- and 14-day induction was further analyzed using quantitative real-time polymerase chain reaction (qRT-PCR). The focus was placed on fibrosis-related type I collagen (*Col I*), hypertrophy-related type X collagen (*Col X*), and chondrocyte-specific markers, including aggrecan (*ACAN*), *Col II*, and SRY-box transcription factor 9 (*SOX9*) (Fig. 3c). Consistent with findings above, *ACAN* mRNA expression was significantly higher in the DSRG and DSRGT groups than in the other groups on days 7 and 14. *Col II* mRNA expression was also markedly elevated in the DSRT and DSRGT groups, whereas *Col I* and *Col X* mRNA levels remained low. This result was attributed to TD-198946, which effectively inhibited fibrosis and hypertrophy during chondrogenic differentiation. Notably, *SOX9* mRNA expression in the DSRGT group increased significantly by day 14, reaching the highest level among all groups. These findings demonstrated that DSRGT effectively promoted the expression of cartilage-specific genes while inhibiting the transition to fibrotic or calcified cartilage. The protein expression of ACAN, Col II, and SOX9 after 14 d of induction was finally evaluated using Western blotting analysis. The results revealed that, compared with the other groups, the DSRGT group presented the highest protein expression levels of ACAN, Col II, and SOX9 (Fig. 3d). These results indicated that while all the groups exhibited some degree of chondrogenic induction, DSRGT demonstrated the most pronounced effects. Therefore, DSRGT was selected as the scaffold material for CO culture and was used for subsequent experiments.

### Evaluation of COs

**Fig. 4:**
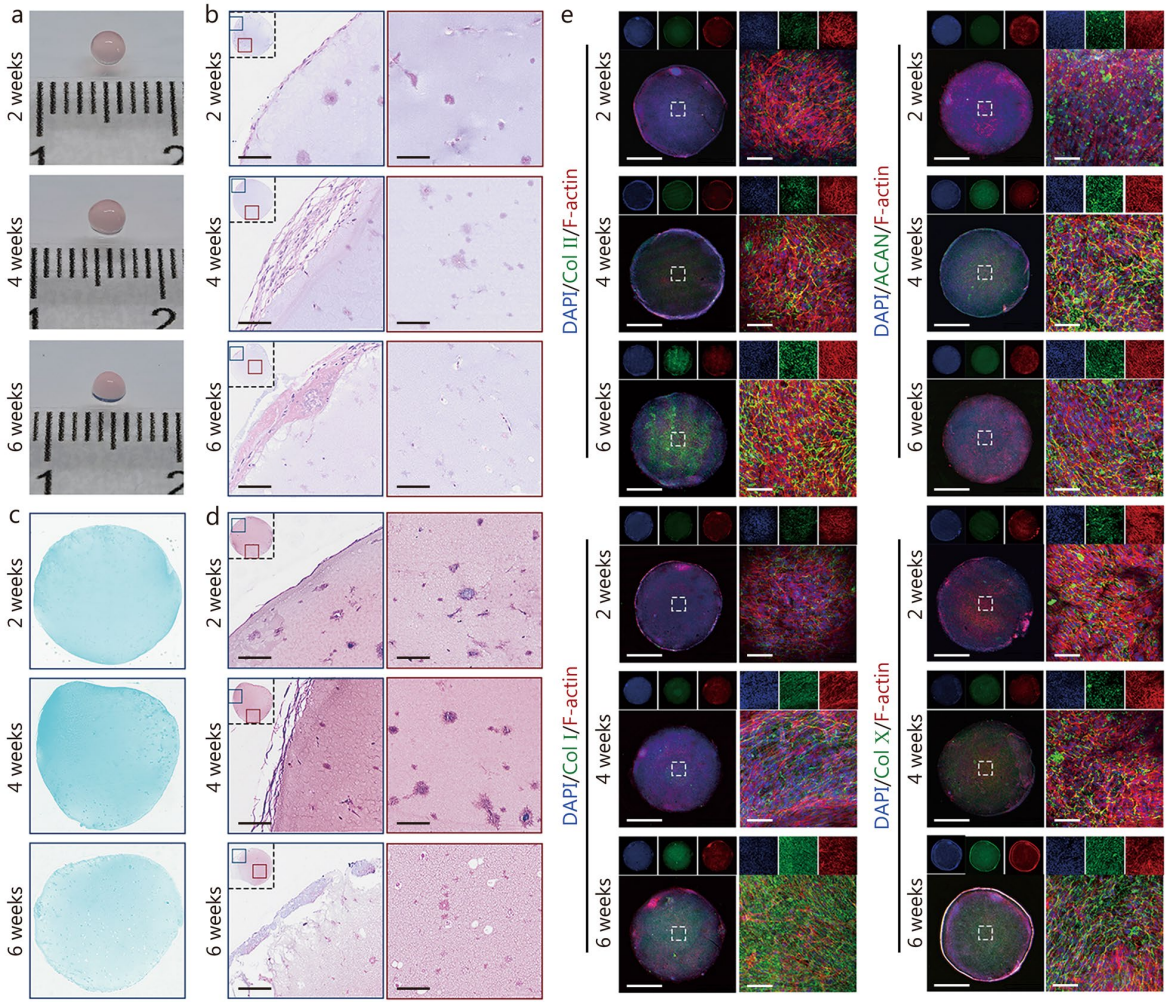
Evaluation of COs cultured for 2, 4, and 6 weeks. **a** Macroscopic images of COs cultured for 2, 4, and 6 weeks. **b** Hematoxylin and eosin (HE) staining results. Scale bar = 100 µm. **c** Alcian blue staining results. **d** Safranin O/Fast Green staining results. Scale bar = 100 µm. **e** Immunofluorescence (IF) results for Col II, ACAN, Col I, and Col X (*n* = 6). Scale bar = 1 nm (left) and 200 µm (right). Data are presented as mean ± SD. One-way ANOVA and Tukey’s multiple-comparisons test were used for data analysis. Col II type II collagen, ACAN aggrecan, Col I type I collagen, Col X type X collagen, DAPI 4′,6-diamidino-2-phenylindole

DSRGT was selected as the scaffold material for constructing COs, which were cultured in chondrogenic induction medium for 6 weeks. To investigate the early chondrogenic potential of COs, constructs cultured for 1 week were first examined. Macroscopic observation showed no significant morphological changes after 1 week of culture (Additional file 1: Fig. S5a). Given that the aggregation of cartilage progenitor cells typically occurs at an early stage, immunofluorescence (IF) staining for SOX9, an early cartilage-specific marker, was performed. Strong SOX9 expression was observed in COs at the 1-week time point, indicating early chondrogenic commitment (Additional file 1: Fig. S5b). Subsequently, qRT-PCR was conducted to analyze gene expression profiles of BMSCs (control) and COs cultured under the same conditions for 1 week (Additional file 1: Fig. S5c). The results showed that the expression levels of chondrogenic markers, including *SOX9*, *Col II*, and *ACAN,* were significantly higher in COs compared to the control group. In contrast, no significant differences were observed in the expression of fibrosis-related *Col I* and hypertrophy-associated *Col X* between the two groups. COs at 2, 4, and 6 weeks were systematically evaluated. Macroscopic observations revealed that the organoids gradually collapsed with prolonged culture time, likely due to the gradual degradation of the DSRGT hydrogel during long-term culture (Fig. 4a).

To further assess morphological changes, COs at different time points were sectioned and stained with hematoxylin and eosin (HE), Alcian blue, and Safranin O/Fast Green. HE staining revealed that at 2 weeks, the surface cell layer of the organoids was relatively dense. By 4 weeks, the cells expanded on the surface, forming multilayered structures. At 6 weeks, the organoid surface exhibited tissue-like structures (Fig. 4b). Within the organoids, cells were uniformly distributed in the hydrogel scaffold. At 2 and 4 weeks, the cells formed tissue-like structures within the hydrogel. However, by 6 weeks, vacuoles caused by cell apoptosis were observed inside the hydrogel. This may have been due to the thickening of the surface cell layer, which hindered nutrient exchange between the internal and external regions. Furthermore, DSRGT had nearly completed drug release by 4 weeks, failing to provide sufficient nutritional support for the internal cells, potentially contributing to vacuole formation. Alcian blue staining revealed that the staining intensity of COs was higher at 4 weeks than at 2 and 6 weeks, indicating that the GAG content was highest at 4 weeks (Fig. 4c). Safranin O/Fast Green staining revealed cartilage-specific red staining in the organoids at 2, 4, and 6 weeks, confirming the successful formation of COs. The staining intensity was stronger at 4 weeks than at 2 and 6 weeks, indicating that the phenotype of the organoids at 4 weeks most closely resembled that of cartilage (Fig. 4d). IF staining was conducted to analyze the expression of cartilage-specific markers (Col II and ACAN), as well as markers related to fibrosis (Col I) and hypertrophy (Col X), in the COs at 2, 4, and 6 weeks (Fig. 4e). The results revealed that Col II expression increased progressively with culture time, peaking at 6 weeks (Additional file 1: Fig. S5d). However, Col I and Col X expression also peaked at 6 weeks. In contrast, at 2 and 4 weeks, Col X expression remained relatively low. This may be attributed to the effects of TD-198946, which effectively inhibited hypertrophy and fibrosis. After 4 weeks, the complete release of TD-198946 led to a significant increase in Col I and Col X expression. Furthermore, ACAN expression was highest at 4 weeks, which corresponded to the near-complete release of Glu by that time. Overall, the phenotype of COs at 4 weeks most closely resembled that of hyaline cartilage, demonstrating the most favorable cartilage-specific characteristics. Therefore, a 4-week culture period appeared to be the optimal time point for CO development, exhibiting the highest potential for achieving the desired cartilage phenotype.

### RNA sequencing of BMSCs in COs cultured for 0, 2, 4, and 6 weeks

**Fig. 5:**
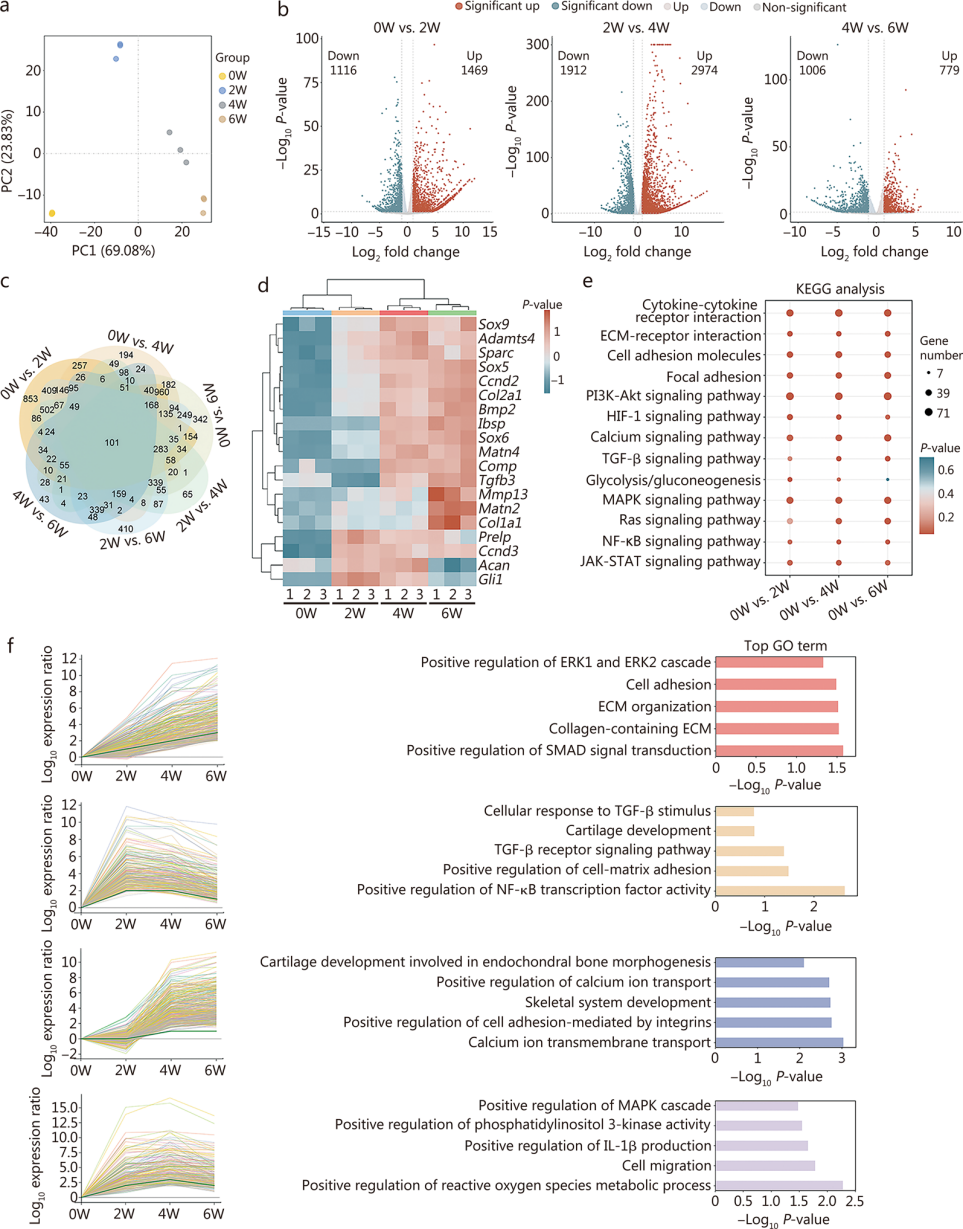
Transcriptomic analysis of BMSCs in COs cultured for 0, 2, 4, and 6 weeks. **a** Principal component analysis (PCA) of differentially expressed genes (DEGs) in COs cultured for 0, 2, 4, and 6 weeks (*n* = 3). **b** Volcano plots of DEGs between 0 and 2W, 2W and 4W, and 4W and 6W. The X- and Y-axes represent the log_2_ fold change and log_10_ *P*-value, respectively. Genes with a log_2_ fold change > 1 and *P_*adjust < 0.05 are considered significantly upregulated, while genes with a log_2_ fold change <  −1 and *P_*adjust < 0.05 are considered significantly downregulated. **c** Venn diagram showing overlapping DEGs across 0W, 2W, 4W, and 6W. **d** Heatmap illustrating the transcriptional levels of key mRNAs regulating chondrogenic development in COs (relative expression value for each gene). **e** KEGG enrichment analysis of upregulated genes in each group. **f** GO enrichment and STEM analysis of upregulated genes in each group. 0W BMSCs from COs cultured for 0 week, 2W BMSCs from COs cultured for 2 weeks, 4W BMSCs from COs cultured for 4 weeks, 6W BMSCs from COs cultured for 6 weeks, COs cartilage organoids, ECM extracellular matrix, PI3K-Akt phosphoinositide 3-kinase-protein kinase B, HIF-1 hypoxia-inducible factor 1, TGF-β transforming growth factor-beta, MAPK mitogen-activated protein kinase, JAK-STAT Janus kinase-signal transducer and activator of transcription, ERK extracellular signal-regulated kinase, SMAD Sma and Mad-related protein, KEGG Kyoto Encyclopedia of Genes and Genomes, GO Gene Ontology, STEM short time-series expression miner, BMSCs bone-marrow mesenchymal stem cells

To comprehensively analyze the differentiation process of COs, high-throughput mRNA sequencing was performed on BMSCs from COs cultured for 0, 2, 4, and 6 weeks (0W, 2W, 4W, and 6W). PCA was performed to compare gene expression patterns across different time points, with the close clustering of points indicating higher similarity in gene expression profiles. The results showed that gene expression profiles were significantly different among the 0W, 2W, 4W, and 6W groups, whereas they were similar within each group (Fig. 5a). The number of significantly DEGs exhibited an initial increase followed by a subsequent decrease, from 2585 DEGs between the 0W and 2W groups to 4886 DEGs between the 2W and 4W groups, and 1785 DEGs between the 4W and 6W groups (Fig. 5b). These results indicated that the most pronounced gene expression changes occurred during the early differentiation stage (2–4 weeks). Further analysis revealed that during the late differentiation stage (4–6 weeks), the number of significantly downregulated DEGs (*n* = 1006) was higher than the number of significantly upregulated DEGs (*n* = 779). Venn diagram analysis identified 311 DEGs consistently expressed across all stages of CO development. Additionally, 1796 shared DEGs were identified across all time points compared with the 0W group (Fig. 5c). To visualize the expression patterns of chondrogenesis-related DEGs, a heatmap was generated, illustrating their dynamic changes during CO culture (Fig. 5d). Chondrogenic differentiation-related genes, including *Sox9*, *Adamts4*, *Sox5*, *Col2a1*, *Matn2*, *Comp*, and *Tgfb3*, were progressively upregulated during the first 4 weeks and either stabilized or further increased at 6 weeks. However, genes associated with cartilage fibrosis (such as *Sparc* and *Col1a1*) and hypertrophy (e.g., *Ibsp*, *Bmp2*, *Mmp13*, and *Matn4*) presented the highest expression levels at 6 weeks. Additionally, the key chondrogenic gene *Acan* and the proliferation-related gene *Gli1* remained stable during the first 4 weeks but decreased significantly by week 6. These findings indicated that COs exhibited trends of fibrosis and hypertrophy after 6 weeks of culture. KEGG pathway enrichment analysis revealed the functional roles of the upregulated DEGs during CO development (Fig. 5e). Pathways such as the cytokine-cytokine receptor interaction pathway, the mitogen-activated protein kinase (MAPK) signaling pathway, the PI3K-Akt signaling pathway, and the calcium signaling pathway exhibited stable expression patterns throughout the culture period. In contrast, the TGF-β signaling pathway and Ras signaling pathway were significantly upregulated after 4 weeks. The glycolysis/gluconeogenesis pathway, which is associated with cartilage differentiation, was not significantly regulated at 6 weeks.

STEM analysis was subsequently performed based on GO enrichment results to explore the dynamic functional changes during organoid culture (Fig. 5f). Functions such as positive regulation of Sma and Mad-related protein (SMAD) signal transduction, collagen-containing ECM, and ECM organization gradually increased over time, indicating their critical roles in CO development. Additionally, calcium ion transmembrane transport and positive regulation of cell adhesion mediated by integrins were upregulated starting at 4 weeks and remained elevated through 6 weeks. In contrast, functions such as positive regulation of NF-κB transcription factor activity, positive regulation of cell–matrix adhesion, and the TGF-β receptor signaling pathway were significantly upregulated at 2 weeks, stabilized at 4 weeks, and decreased significantly at 6 weeks. Notably, the positive regulation of reactive oxygen species metabolic process and cell migration reached their highest expression levels at 4 weeks.

In conclusion, the results of the mRNA sequencing analysis identified 4 weeks as a critical time point for CO development. Therefore, 4-week-cultured COs were selected for subsequent in vivo validation experiments.

### In vivo evaluation of cartilage repair using COs

**Fig. 6:**
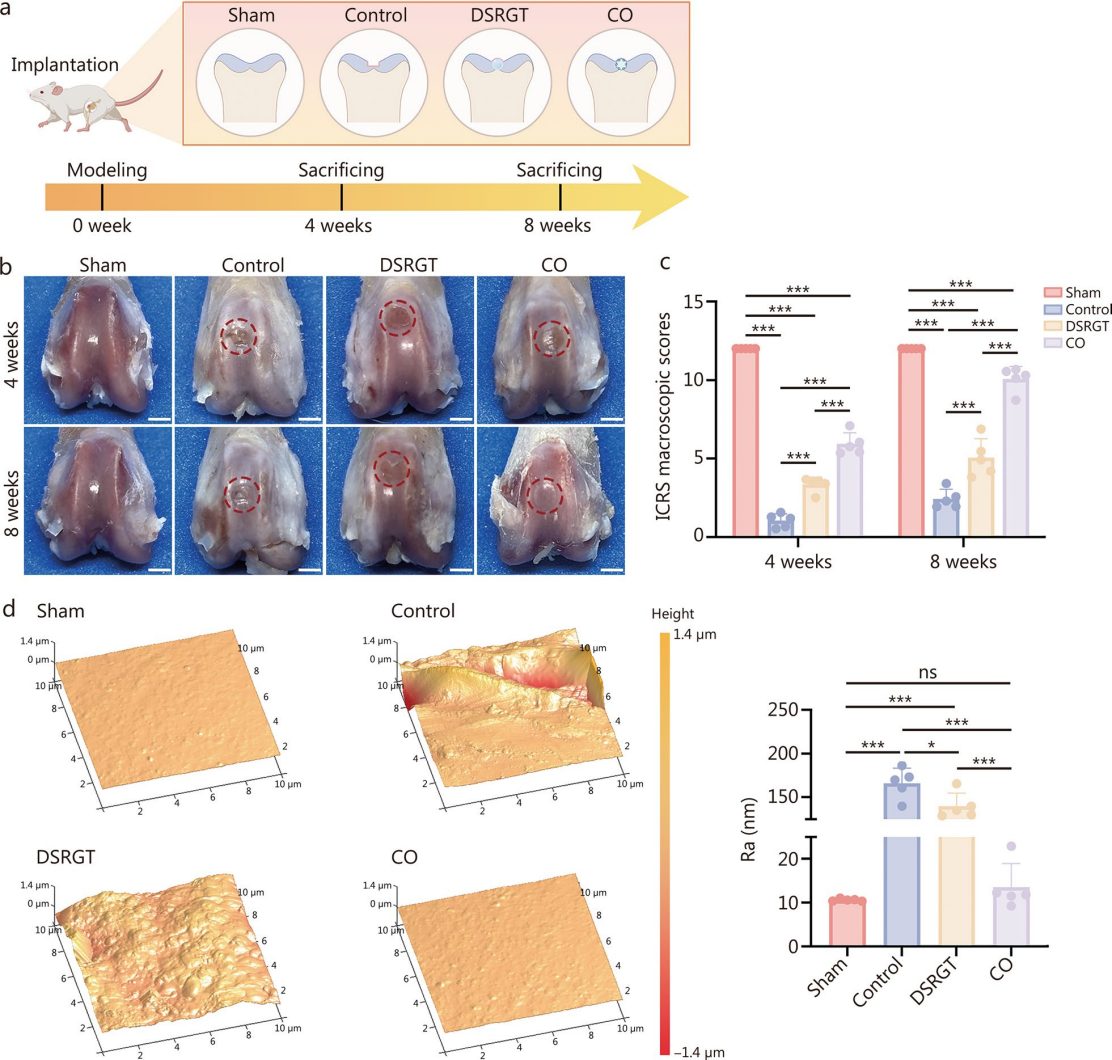
Macroscopic evaluation of regenerated cartilage at 4 and 8 weeks post-treatment. **a** Schematic representation of defect creation and experimental grouping. **b** Macroscopic images of cartilage defects at 4 and 8 weeks post-treatment. Scale bar = 2 mm. **c** ICRS scoring results of the 4 groups (*n* = 5). **d** AFM images and Ra of regenerated cartilage surfaces at 8 weeks post-treatment. Data are presented as mean ± SD. One-way ANOVA and Tukey’s multiple-comparisons test were used for data analysis. *P < 0.05, ***P < 0.001, ns non-significant. Sham positive control, Control untreated defects covered with fibrin glue, DSRGT defects treated with DSRGT and fibrin glue, CO defects treated with 4-week cultured cartilage organoids and fibrin glue, ICRS International Cartilage Repair Society, Ra roughness, AFM atomic force microscopy

As illustrated in Fig. 6a, the regenerative potential of COs cultured for 4 weeks was evaluated in a rat articular cartilage defect model (defect size: 0.2 mm × 0.2 mm).

Macroscopic evaluation of full-thickness cartilage repair at the distal femur revealed significant differences among groups (Fig. 6b). The cartilage surface in the sham group was smooth and homogeneous. The control group exhibited irregular cartilage repair with pitted surfaces, blurred defect edges, and degenerative changes in the surrounding cartilage. The DSRGT group showed smoother and more uniform repair tissue, but with reduced thickness. The CO group demonstrated the best repair outcomes, with well-filled defects, smooth surfaces, and no degeneration of the surrounding cartilage, resembling native cartilage morphology. Macroscopic assessment using the International Cartilage Repair Society (ICRS) scoring system (Fig. 6c) revealed the poorest repair outcomes in the control group. The CO group significantly outperformed both the control and DSRGT groups, achieving the highest repair scores by week 8. Compared with the control group, the DSRGT group also exhibited significantly improved repair. All groups exhibited progressively higher ICRS scores over time, reaching a maximum score of 10.08 ± 0.79 at week 8. To further evaluate the surface smoothness of regenerated cartilage, atomic force microscopy (AFM) was used to measure surface roughness (Ra) parameters at week 8 (Fig. 6d). The Ra values in the CO group were slightly higher than in the sham group but the difference was not statistically significant, and these values were significantly lower than those of the control and DSRGT groups.

To quantitatively evaluate the quality of tissue repair, HE staining, Safranin O/Fast Green staining, and immunohistochemical (IHC) staining for Col II and ACAN were performed. HE and Safranin O/Fast Green staining revealed significant differences in repair quality among the groups (Additional file 1: Fig. S6a, b). In the sham group, a healthy cartilage structure was observed, characterized by a smooth surface, a cartilage matrix layer approximately 200 μm thick, and chondrocytes predominantly distributed near the surface of the cartilage layer. In the control group, severe cartilage damage was observed as early as week 4, with ECM deposition and repair tissue predominantly composed of fibrous tissue. The regenerated tissue exhibited uneven cellular distribution and degeneration of the surrounding cartilage. By week 8, although the regenerated cartilage had thickened, its structure remained irregular, with minimal matrix remodeling. Some areas of the lower cartilage had undergone ossification, and the cell density was significantly reduced and unevenly distributed. In the DSRGT group, partial filling and regeneration of the cartilage defect were observed at week 4, but the surface structure was irregular, and matrix staining was inconsistent. By week 8, the regenerated cartilage had become flush with the surrounding cartilage, with a relatively uniform cell distribution. However, the matrix remained unstained, indicating that the repair tissue primarily consisted of fibrous tissue. In contrast, the CO group exhibited significantly better outcomes. At 4 weeks, the CO group displayed regenerated cartilage that was flush with the surrounding tissue, with distinct cartilage matrix staining, indicating the regeneration of a substantial amount of cartilage matrix and high cell density. Over time, the staining intensity and thickness of the cartilage matrix gradually increased. By week 8, the regenerated cartilage in the CO group exhibited a smooth surface comparable to that of the sham group. However, unlike native cartilage, which shows a gradual decrease in cell density from the surface to deeper layers, the regenerated cartilage in the CO group presented relatively uniform cell density. Additionally, while cartilage matrix staining in natural cartilage gradually weakens from surface to deep layers, the CO group exhibited the opposite trend. This may be due to the even distribution of cells within the implanted COs, with surface cells secreting the most ECM. Future studies are needed to develop COs that can mimic the layered characteristics of articular cartilage. The O’Driscoll scoring system was used to quantitatively assess the extent and quality of cartilage repair (Additional file 1: Fig. S7a). The results revealed that the CO group consistently achieved significantly higher scores than did the control and DSRGT groups at all time points. By week 8, the histological scores of the CO group had approached those of the sham group.

The IHC staining and semiquantitative analysis results were consistent with the Safranin O/Fast Green staining results, which revealed that the expression levels of Col II and ACAN in the CO group were significantly higher than those in the control and DSRGT groups at all time points (Additional file 1: Fig. S6c, d; Fig. S7b). At 8 weeks, Col II immunostaining in the CO group was primarily localized in the subchondral layer, similar to that in the sham group. ACAN secretion was higher in the CO group than in the sham group, although the difference was not statistically significant, possibly due to the higher number of newly formed chondrocytes in the CO group. Overall, these findings indicated superior cartilage repair in the CO group. Furthermore, the in vivo toxicity of the implanted materials and COs was assessed. HE staining revealed that the major organ anatomy of the rats was normal at 8 weeks post-surgery (Additional file 1: Fig. S8a). Routine blood analysis and liver and kidney function tests revealed no significant systemic toxicity in any group (Additional file 1: Fig. S8b), further confirming the safety of both DSRGT and CO in vivo.

### Mechanisms underlying cartilage regeneration by COs

**Fig. 7:**
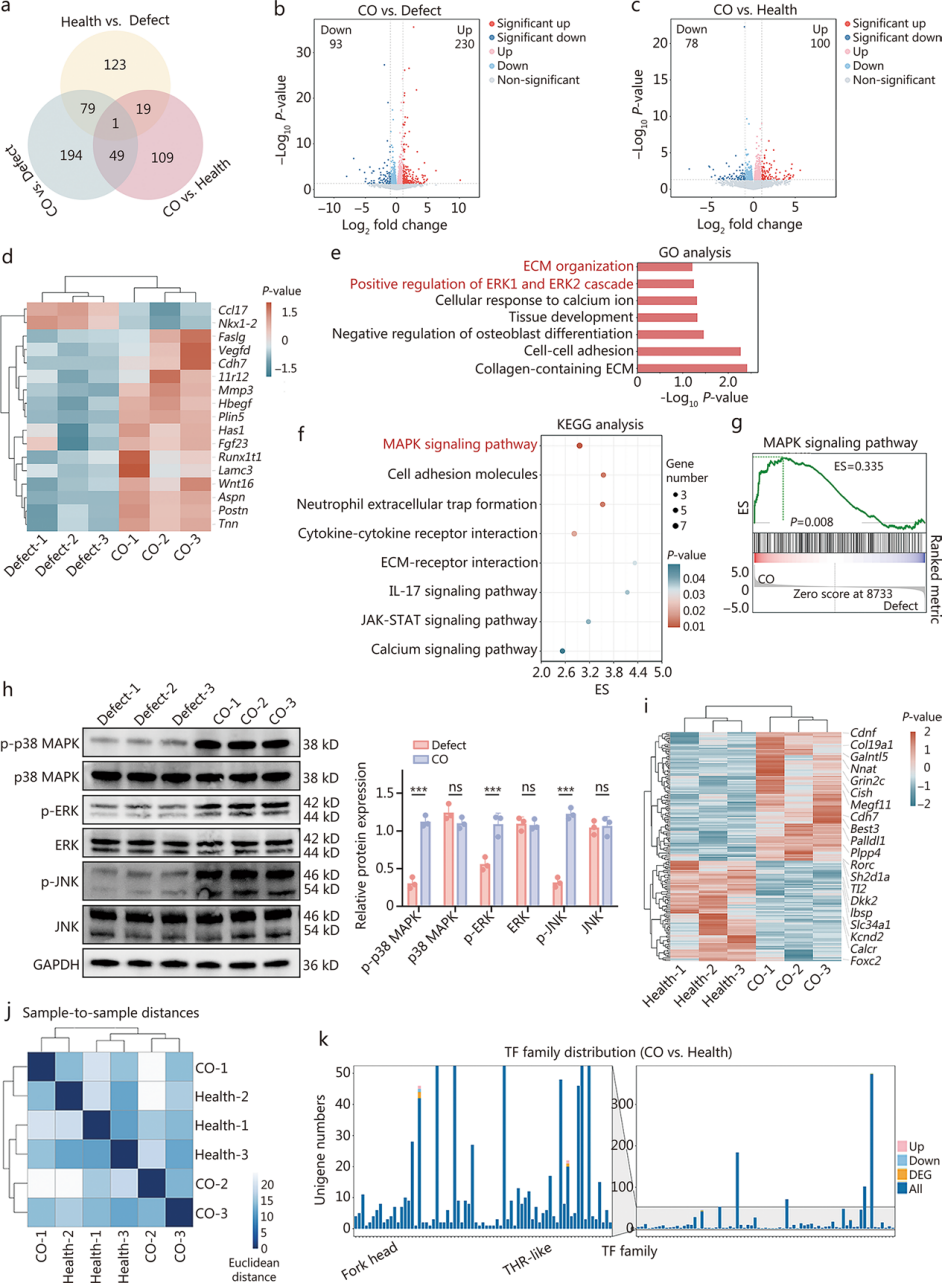
mRNA analysis of regenerated cartilage from the defect group, CO group, and healthy group. **a** Venn diagram. **b** Volcano plot comparing DEGs between regenerated cartilage from the defect group and the CO group. The X- and Y-axes represent the log_2_ fold change and log_10_ *P*-value, respectively. Genes with a log_2_ fold change > 1 and *P*_adjust < 0.05 are considered significantly upregulated, while genes with a log_2_ fold change < −1 and *P*_adjust < 0.05 are considered significantly downregulated. **c** Volcano plot comparing DEGs between the CO group and the healthy group. The X- and Y-axes represent the log_2_ fold change and log_10_ *P*-value, respectively. Genes with a log_2_ fold change > 1 and *P*_adjust < 0.05 are considered significantly upregulated, while genes with a log_2_ fold change <  −1 and *P*_adjust < 0.05 are considered significantly downregulated. **d** Heatmap of key mRNA transcripts associated with cartilage regeneration, comparing the defect group and the CO group (relative expression value for each gene). **e** GO analysis of upregulated genes between the defect group and the CO group. **f** KEGG pathway analysis of upregulated genes between the defect group and the CO group. **g** Gene set enrichment analysis (GSEA) results comparing regenerated cartilage from the defect group and the CO group. **h** Western blotting and quantitative analysis for the defect group and the CO group (*n* = 3). **i** Heatmap of key mRNA transcripts associated with cartilage regeneration, comparing regenerated cartilage from the CO group and the healthy group (relative expression value for each gene). **j** Sample-to-sample clustering analysis comparing gene expression profiles between the CO group and the healthy group. **k** Family distribution analysis of TFs between the CO group and the healthy group. Data are presented as mean ± SD. One-way ANOVA and Tukey’s multiple-comparisons test were used for data analysis. ***P < 0.001, ns non-significant. Defect regenerated cartilage from the untreated group after 8 weeks, CO regenerated cartilage from the CO group after 8 weeks, Health regenerated cartilage from healthy cartilage, ECM extracellular matrix, ERK extracellular signal-regulated kinase, MAPK mitogen-activated protein kinase, IL-17 interleukin-17, JAK-STAT Janus kinase-signal transducer and activator of transcription, ES enrichment score, p-p38 MAPK phosphorylated mitogen-activated protein kinase p38, p38 MAPK mitogen-activated protein kinase p38, p-ERK phosphorylated extracellular signal-regulated kinase, p-JNK phosphorylated c-Jun N-terminal kinase, JNK c-Jun N-terminal kinase, GAPDH glyceraldehyde-3-phosphate dehydrogenase, TF transcription factor, DEG differentially expressed gene, GO Gene Ontology, KEGG Kyoto Encyclopedia of Genes and Genomes, THR thyroid hormone receptor

To comprehensively investigate the role of COs as grafts in cartilage defect repair, high-throughput mRNA sequencing was performed on regenerated cartilage from the untreated group after 8 weeks (damaged cartilage), regenerated cartilage from the CO group after 8 weeks, and healthy cartilage. Venn diagram analysis revealed 80 DEGs shared between the CO group and healthy cartilage compared with damaged cartilage. Additionally, 20 DEGs were shared between the CO group and damaged cartilage when compared with healthy cartilage, indicating that the gene expression profile of the CO group more closely resembles that of healthy cartilage than that of damaged cartilage (Fig. 7a). Specifically, compared with damaged cartilage, the CO group presented 230 significantly upregulated genes and 93 significantly downregulated genes (Fig. 7b; Additional file 1: Fig. S9a). Compared with healthy cartilage, the CO group presented 100 upregulated genes and 78 downregulated genes (Fig. 7c). To visualize the differences between regenerated cartilage in the CO group and damaged cartilage, a heatmap of genes associated with cartilage regeneration was generated, which revealed more pronounced upregulation of these genes in the CO group (Fig. 7d). GO analysis revealed that the regenerated cartilage in the CO group presented upregulated pathways associated with cartilage matrix synthesis, such as collagen-containing ECM and ECM organization (Fig. 7e). Notably, the regenerated cartilage also upregulated the positive regulation of the extracellular signal-regulated kinase 1 (ERK1) and ERK2 cascades, with ERK being a key member of the MAPK family. Correspondingly, KEGG analysis revealed that the regenerated cartilage in the CO group also upregulated the MAPK signaling pathway (Fig. 7f). Gene set enrichment analysis (GSEA) and pathway gene heatmap results further supported these findings (Fig. 7g; Additional file 1: Fig. S9b). Previous studies have confirmed that the activation of ERK and p38 in the MAPK signaling pathway promotes the chondrogenic differentiation of MSCs [31, 32]. Under stress conditions, the activation of c-Jun N-terminal kinase (JNK) promotes chondrocyte differentiation and the expression of cartilage-specific genes [33]. To verify this process, the activation of the MAPK signaling cascade, including ERKs, p38 kinase, and JNKs, was assessed. As shown in Fig. 7h, the levels of p-p38, p-ERK, and p-JNK were significantly elevated in the CO group. These results suggest that cartilage regeneration was promoted in the CO group via the upregulation of the MAPK signaling pathway. To visually compare the differences in gene expression between the CO group and the healthy group, a clustered heatmap of DEGs was generated, with a focus on genes related to cartilage regeneration (Fig. 7i). In addition, PD98059 was added during in vitro culture to inhibit the ERK pathway. Western blotting analysis was conducted on the control group (uninduced BMSCs), the PD98059 group (COs cultured with PD98059 for 4 weeks), and the CO group (COs cultured for 4 weeks). The results demonstrated that inhibition of the ERK-MAPK pathway significantly suppressed the expression of chondrogenic differentiation markers (ACAN, Col II), thereby impairing COs development (Additional file 1: Fig. S9c). Sample clustering analysis between the CO and healthy groups revealed a low clustering coefficient. Some samples from the CO and healthy groups were classified into the same cluster, indicating a relatively similar gene expression profile between them (Fig. 7j). However, the long-term effects on joint mechanics and integration remain uncertain. TF family distribution analysis revealed differential expression in only 3 TF families, namely, zfC2H2, thyroid hormone receptor (THR)-like, and Forkhead (Fig. 7k). Notably, Forkhead TFs play crucial roles in cartilage development. By interacting with other key TFs, they regulate chondrocyte differentiation, proliferation, and maturation. Specifically, Forkhead box C and Forkhead box D are involved in chondroprogenitor differentiation and act synergistically with SOX9 to regulate chondrocyte differentiation, which is critical for cartilage formation and repair. On the other hand, THR-like TFs tended to be upregulated, likely due to their association with THRs, which are involved in regulating cell proliferation, differentiation, and metabolism. Compared with stable healthy cartilage, the CO group exhibited more upregulated signaling pathways related to these processes to facilitate cartilage repair. Overall, the implantation of COs in the CO group promoted cartilage regeneration by upregulating the MAPK signaling pathway.

## Discussion

Cartilage injury affects millions of individuals worldwide, making cartilage repair a critical focus in regenerative medicine [34, 35]. Compared with the repair process using traditional biomaterials, COs bypass the need for endogenous cell recruitment and proliferation, significantly shortening the repair timeline. Consequently, constructing COs with native cartilage-like properties has emerged as a research priority.

The scaffold-free self-organization method relies on the self-aggregation and differentiation capacities of stem or progenitor cells, enabling the formation of 3D organoids without external scaffolds. This method has been widely applied in CO research, yielding important advances. For example, Donges et al. [36] utilized human BMSCs to construct COs cultured for 28 d. By applying inflammatory stimuli at specific stages of chondrogenesis, they developed a CO model that mimicked key pathological features of human OA, including chondrocyte hypertrophy, cartilage matrix mineralization, enhanced catabolism, and mechanical stiffening. Wei et al. [37] employed clustered regularly interspaced short palindromic repeats (CRISPR)–CRISPR-associated protein 9 (Cas9) gene-editing technology to label human extended pluripotent stem cells with the dual reporter genes, *COL2A1* (collagen type II alpha 1)-mCherry and *COL10A1* (collagen type X alpha 1)-enhanced green fluorescent protein. This approach enabled the real-time monitoring of chondrogenesis and hypertrophy during CO formation, providing a valuable tool for identifying therapeutic targets for cartilage regeneration. Additionally, Huo et al. [38] formed COs by suspending chondrocytes to create cell aggregates, which self-assembled into spherical structures in culture dishes. These COs regulated cell aggregation through cell adhesion proteins and cytoskeletal remodeling. After preculture, the COs were implanted into immunodeficient mice, where in vivo culture facilitated their fusion into regenerative cartilage with specific 3D shapes and functional properties. Notably, precultured autologous COs were injected into goats, successfully achieving nasal dorsum augmentation. These findings highlight the potential application of organoid-based tissue engineering technologies in plastic and reconstructive surgeries for clinical translation. Hyaline cartilage is an avascular tissue primarily composed of water, Col II, and ACAN, with chondrocytes being the sole cell type, accounting for only 1–2% of its total volume [39]. However, COs generated using scaffold-free self-organization typically exhibit a much higher cell density than natural cartilage does, leading to significant differences in composition and structure.

As another strategy, coculture with biomaterials provides physical support for COs, enabling precise regulation of cell density and spatial distribution to better mimic the composition and structure of native cartilage [40]. Biomaterials were also found to simulate the ECM microenvironment, allowing precise control over cell behavior through their mechanical properties, degradability, and bioactivity [41–43]. This property facilitated the formation of COs with more physiologically relevant structures. Additionally, biomaterials can incorporate growth factors or drugs to further promote cell proliferation and differentiation, enhancing the functionality and bioactivity of organoids. For example, Kronemberger et al. [44] developed an oxidized alginate hydrogel bioink with rapid degradation properties (full degradation within 2–3 weeks). This bioink, combined with cartilage microtissues, was used in 3D bioprinting to construct cartilage grafts with excellent biocompatibility and mechanical support. As supporting material, oxidized alginate hydrogels demonstrated multiple advantages in cartilage tissue engineering, including facilitating cell fusion, improving biocompatibility, enhancing nutrient exchange, regulating cell metabolism, and providing mechanical support. Jia et al. [45] utilized 3D bioprinting and a microporous photocurable decellularized cartilage matrix-based bioactive bioink to fabricate auricular cartilage regeneration scaffolds with precise shapes and superior mechanical strength. These novel scaffolds enabled the regeneration of anatomically accurate, elastic, and cartilage-specific ECM-deposited auricular cartilage in immunodeficient mice. In addition, Wang et al. [46] integrated 3D bioprinting with siRNA, aynovium-derived mesenchymal stem cells-derived organoid, and bioink to construct a novel organoid hydrogel polymer composite scaffold. This scaffold demonstrated superior hyaline cartilage characteristics when subcutaneously implanted. These findings highlight the key advantages of biomaterial-based coculture methods in CO construction, providing essential technological support for regenerative medicine and cartilage repair.

In vivo organoid construction approaches, such as subcutaneous transplantation, typically provide a blood supply to implants. However, hyaline cartilage is an avascular tissue, making subcutaneous transplantation unsuitable for cultivating hyaline COs. In contrast, in vitro cultivation of COs circumvents the blood supply issue. Matrigel is widely used as a biomimetic ECM for organoid construction. However, its insufficient mechanical strength and undefined composition limit its application in hyaline CO development. Replacing Matrigel with alternative biomaterials has become a critical focus in advancing CO research [47]. Recent studies have explored various novel materials suitable for CO construction. For example, Zhao et al. [48] proposed a “molecular Velcro” strategy. They coassembled the amphiphilic tripeptide 4-biphenylalanine-glycine-phenylalanine-phenylalanine with gelatin methacrylate. This hydrogel, formed via intermolecular interactions and exhibited increased mechanical strength and bioactivity. The coassembled hydrogel, loaded with BMSCs, was photopolymerized and stably fixed into rabbit knee joint defects. After 3 months, the hydrogel significantly promoted hyaline cartilage regeneration. Similarly, Ayushman et al. [49] developed a “cell rolling” strategy by designing PEG-based sliding hydrogels. These hydrogels utilize movable cross-links and ligand features to enhance the physical interaction between cells and the hydrogel microenvironment. This approach significantly promoted MSCs differentiation into chondrocytes. The mechanism involves cytoskeletal and nuclear activity enhancement, nuclear mechanotransduction, and chromatin state alteration. In addition, Yang et al. [50] combined hyaluronic acid and hydroxyapatite with gelatin-based microcrystalline gels. These were coassembled via intermolecular interactions to form customized chondrogenesis-microcryogels and osteogenesis-microcryogels with cartilage and bone regeneration capacity. These microgels self-assembled in situ with MSCs, creating osteochondral organoids capable of both cartilage and bone regeneration. Notably, superior cartilage repair was achieved with organoids compared to MSC-loaded microcryogels.

Currently, hydrogel scaffolds capable of supporting long-term in vitro culture of COs are lacking. This study developed DSRGT by covalently grafting Glu and TD-198946 onto a DNA-SF hydrogel network modified with AC-PEG-NHS. Glu, as a precursor of the cartilage matrix, plays a critical role in cartilage regeneration. A previous study demonstrated that the grafting of Glu onto hydrogels significantly promoted the chondrogenesis of BMSCs [51]. Furthermore, the transplantation of Glu-modified hydrogels with BMSCs resulted in cartilage-like tissue formation within 8 weeks. TD-198946, known for its chondrogenic properties, has garnered considerable attention since its discovery. One study has shown that coculture of TD-198946 with chondrocytes results in the production of cell sheets with enhanced cartilage regeneration capabilities [52]. These cell sheets, when transplanted to cartilage defect sites, effectively facilitated cartilage repair. In previous studies, a DNA-SF hydrogel was successfully developed, which promoted the chondrogenic differentiation of BMSCs through surface stiffness [21]. The DNA formed a supramolecular network via base pairing, constraining and aggregating silk fibroin molecules, thus inducing the development of β-sheet structures. Subsequently, covalent grafting of Glu and TD-198946 was introduced to enhance its properties. DSRGT not only promoted the chondrogenic differentiation of BMSCs but also inhibited their differentiation toward fibrosis and hypertrophy. Additionally, owing to its sustained-release characteristics, the effect could be maintained for up to 4 weeks, providing a reliable scaffold material for long-term organoid culture, thereby contributing to the optimal maintenance of the hyaline cartilage phenotype at week 4 of culture. DSRGT proved to be an ideal matrix for CO construction and development. When 4-week COs were transplanted into a rat femoral trochlear cartilage defect model, the COs promoted cartilage regeneration by upregulating the MAPK signaling pathway. Cartilage regeneration was achieved within 8 weeks posttransplantation. Gene enrichment analysis revealed that regenerated cartilage in the CO group at 8 weeks presented a gene expression profile relatively similar to that of healthy hyaline cartilage. TF family distribution analysis revealed minimal transcriptional regulatory differences among samples, with variations primarily in the zfC2H2, THR-like, and Forkhead families. The Forkhead family synergized with SOX9 to regulate chondrocyte differentiation and maturation, while THR-like factors modulated cell proliferation, differentiation, and metabolic signaling to promote cartilage repair. These findings demonstrated that CO provided an innovative graft for cartilage defect regeneration.

Several limitations exist in this study. (1) The optimal culture duration in this study was determined based on the drug release time of DSRGT, which does not apply to other CO development cycles. In the future, ultrasound-responsive or infrared-responsive drug release designs will be incorporated into hydrogel systems for cascade drug release, aiming to prolong the sustained release time and explore longer CO developmental cycles. (2) Although gene expression in the newly formed cartilage was measured in this study, the long-term impact on joint integration remains unknown. Future studies could focus on gene expression at the boundary region to reveal the long-term effects of organoids as implants on joint integration. (3) The use of a rat cartilage defect model limits the applicability of this study to human cartilage repair, especially in weight-bearing joints. Future studies will incorporate mechanical loading experiments to assess the load-bearing capacity of COs and their regenerated cartilage, as well as tribological tests under simulated in vivo conditions to evaluate the long-term mechanical integrity and durability. (4) Although DSRGT and COs are biocompatible and safe in a rat model, the human immune system may respond differently. Future experiments should use human-derived cells to obtain more relevant data. (5) The scalability and clinical translation of COs face challenges. Solutions include the use of automation and high-throughput systems to ensure batch uniformity and Raman spectroscopy to monitor the expression of key functional genes and proteins for quality control. Additionally, potential regulatory hurdles must be addressed, including the requirement for COs to meet ISO 10993 standards for implantation therapies.

COs are not limited to use as grafts for cartilage defect repair [53]. They also serve as disease models and drug screening tools, substantially reducing the need for animal experiments [54]. Future efforts could aim to construct calcified COs and OA models based on this study. These models could be used for drug screening and exploration of disease mechanisms. Investigating the developmental patterns of COs in vitro would provide insights into their correlation with in vivo cartilage development, enhancing the understanding of cartilage formation and regeneration processes. The incorporation of responsive properties into biomaterials offers precise control over the developmental direction of COs. This approach expands the potential applications of COs in regenerative medicine, disease modeling, and drug screening.

## Conclusions

**Fig. 8:**
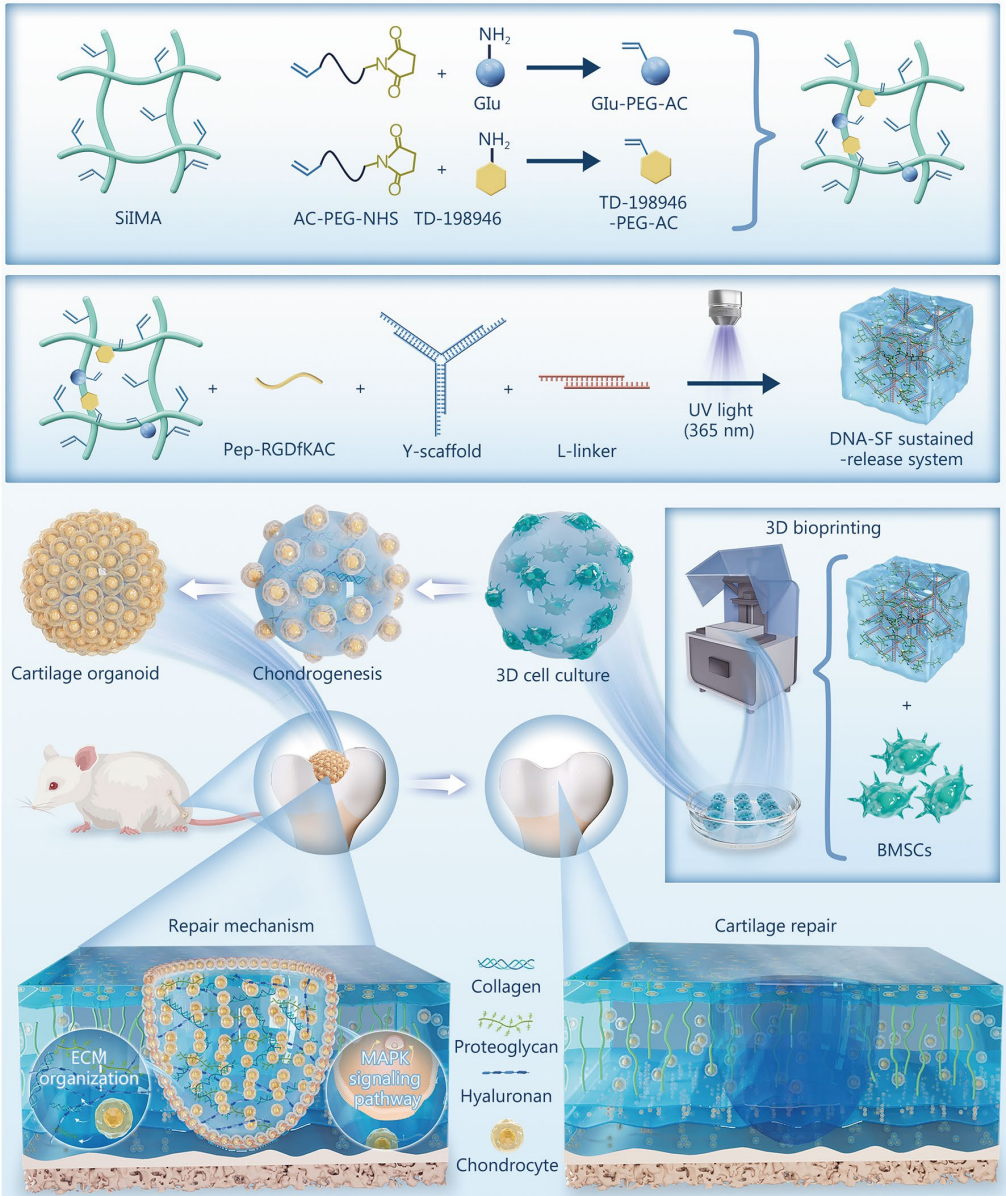
DNA-SF sustained-release system synthesis and its mechanism for cartilage repair. Glu glucosamine, BMSCs bone-marrow mesenchymal stem cells, AC-PEG-NHS acrylic acid-polyethylene glycol-N-hydroxysuccinimide, ECM extracellular matrix, SilMA silk fibroin methacrylate, UV ultraviolet, DNA-SF DNA-silk fibroin, Pep-RGDfKAC RGD-containing peptide modified with D-Phe-Lys and an AC functional group, MAPK mitogen-activated protein kinase

In this study, a novel sustained-release system, DSRGT, was developed to support the long-term culture of COs. Using a DLP system, millimeter-scale COs were successfully fabricated. This strategy promoted the chondrogenic differentiation of BMSCs and inhibited their fibrotic and hypertrophic progression during long-term culture. The developmental process of COs was further investigated, and 4 weeks was identified as the optimal time point for constructing hyaline COs. In addition, transplantation of COs was shown to enhance cartilage regeneration by upregulating the MAPK signaling pathway (Fig. 8). In conclusion, COs constructed with DSRGT constitute a novel and efficient strategy for cartilage defect regeneration.

## Supplementary Information

**Additional file 1: Materials and methods. Table S1** DNA sequence information (the bold sequences are the sticky ends). **Table S2** Antibodies for Western blotting, IF, and IHC. **Table S3** Primer sequences of the genes for qRT-PCR analysis. **Fig. S1** Synthesis and characterization of DSRGT. **Fig. S2** Models 3D-printed using a DLP system based on DSRGT. **Fig. S3** Effects of DSR, DSRG, DSRT, and DSRGT on BMSC biocompatibility and spreading. **Fig. S4** TD-198946 activates the Notch3 signaling pathway in BMSCs. **Fig. S5** Evaluation of COs. **Fig. S6** Histological staining and IHC staining results of regenerated cartilage at 4 and 8 weeks post-treatment. **Fig. S7** Evaluation of regenerated cartilage at 4 and 8 weeks post-treatment. **Fig. S8** In vivo toxicity evaluation. **Fig. S9** Cartilage organoids (COs) promote cartilage regeneration by upregulating the MAPK pathway

## Data Availability

Data supporting the findings of this study can be obtained from the corresponding author upon request.

## Abbreviations

ACAN: Aggrecan
AC-PEG-NHS: Acrylic acid-polyethylene glycol-*N*-hydroxysuccinimide
AFM: Atomic force microscopy
BMP2: Bone morphogenetic protein 2
BMSCs: Bone-marrow mesenchymal stem cells
CCK-8: Cell counting kit-8
COs: Cartilage organoids
Col II: Type II collagen
Col I: Type I collagen
Col X: Type X collagen
3D: Three-dimensional
DEGs: Differentially expressed genes
DNA-SF: DNA-silk fibroin
DLP: Digital light processing
DMMB: 1,9-Dimethylmethylene blue
DSR: RGD-modified DNA-silk fibroin hydrogel
DSRGT: DNA-silk fibroin hydrogel sustained-release system
DSRG: Glu-containing DSR
DSRT: TD-198946-containing DSR
ECM: Extracellular matrix
ELISA: Enzyme-linked immunosorbent assay
ERK: Extracellular signal-regulated kinase
FE-SEM: Field-emission scanning electron microscopy
FTIR: Fourier transform infrared spectroscopy
GAG: Glycosaminoglycan
Glu: Glucosamine
G′: Storage modulus
G″: Loss modulus
GO: Gene ontology
GSEA: Gene set enrichment analysis
HE: Hematoxylin and eosin
ICRS: International Cartilage Repair Society
IHC: Immunohistochemical
JNK: C-Jun N-terminal kinase
KEGG: Kyoto encyclopedia of genes and genomes
LAP: Lithium phenyl-2,4,6-trimethylbenzoylphosphinate
NMR: Nuclear magnetic resonance
OA: Osteoarthritis
PCA: Principal component analysis
qRT-PCR: Quantitative real-time polymerase chain reaction
Ra: Roughness
RGD: Arg-Gly-Asp
SilMA: Silk fibroin methacrylate
SOX9: SRY-box transcription factor 9
STEM: Short time-series expression miner
ssDNA: Single-stranded DNA
TF: Transcription factor
TGF-β3: Transforming growth factor-β3
UV: Ultraviolet
