# Supplementary material for: Accelerating cartilage regeneration with DNA-SF hydrogel sustained release system-based cartilage organoids
Source: Mil Med Res. 2025 Jul 28;12:39. doi: 10.1186/s40779-025-00625-z (PMC12302690; doi:10.1186/s40779-025-00625-z)
Supplement: Supplementary file 1 — Additional file 1: Materials and methods. Table S1 DNA sequence information (the bold sequences are the sticky ends). Table S2 Antibodies for Western blotting, IF, and IHC. Table S3 Primer sequences of the genes for qRT-PCR analysis. Fig. S1 Synthesis and characterization of DSRGT. Fig. S2 Models 3D-printed using a DLP system based on DSRGT. Fig. S3 Effects of DSR, DSRG, DSRT, and DSRGT on BMSC biocompatibility and spreading. Fig. S4 TD-198946 activates the Notch3 signaling pathway in BMSCs. Fig. S5 Evaluation of COs. Fig. S6 Histological staining and IHC staining results of regenerated cartilage at 4 and 8 weeks post-treatment. Fig. S7 Evaluation of regenerated cartilage at 4 and 8 weeks post-treatment. Fig. S8 In vivo toxicity evaluation. Fig. S9 Cartilage organoids (COs) promote cartilage regeneration by upregulating the MAPK pathway [file 40779_2025_625_MOESM1_ESM.pdf]

Materials and methods

Agarose gel electrophoresis

A 2% agarose gel was prepared using agarose (Biowest, Spain) and GelRed dye (Life iLab, China) dissolved in TE buffer. DNA samples were mixed with loading buffer (TaKaRa, Japan) and loaded into the wells. Electrophoresis was performed at 100 V for 60 min. The DNA bands were visualized using an enhanced chemiluminescence detection system.

Nuclear magnetic resonance (NMR)

Glucosamine (Glu) and TD-198946 were reacted with acrylic acid-polyethylene glycol-N-hydroxysuccinimide (AC-PEG-NHS) and fully dissolved. The resulting silk fibroin solutions were transferred to dialysis bags with a molecular weight cut-off of 1000 Da. Dialysis was performed in deionized water for 2 d, with water changes every 8 h to remove unreacted small molecules. After dialysis, the AC-PEG-Glu and AC-PEG-TD-198946 solutions were frozen at –196 °C for 10 min and subsequently freeze-dried for 48 h. Glu and AC-PEG-Glu were dissolved in D_2O, while TD-198946 and AC-PEG-TD-198946 were dissolved in either D_2O or dimethyl sulfoxide (DMSO). NMR spectra were acquired using a JEOL 400 MHz spectrometer (Japan).

Fourier transform infrared spectroscopy (FTIR)

The 4 hydrogel groups were frozen at −196 °C for 10 min and freeze-dried for 48 h. The freeze-dried samples were ground into powder and mixed with KBr to form pellets. FTIR spectroscopy was performed using a Nicolet iS10 spectrometer (Thermo Fisher Scientific, USA) in the range of 500 – 4000 cm^{-1}. Fourier self-deconvolution in the 1600 – 1700 cm^{-1} region was performed using PeakFit software.

Scanning electron microscopy (SEM)

Freeze-dried three-dimensional (3D)-printed spheres or hydrogel surfaces were coated with gold using a high-vacuum ion sputter coater (Leica EM ACE600, Germany). The microstructure and morphology of the samples were observed using a field emission SEM (JSM-7500F, Japan). Pore size and porosity of the freeze-dried hydrogels were quantified from SEM images using ImageJ software.

Rheological properties

The rheological properties of the hydrogels were evaluated using a rheometer (TA HR20, USA). Shear-thinning characteristics and viscosity were measured at 25 °C. Frequency sweeps were performed over a range of 0.1 – 100 rad/s with a constant shear strain of 1%. Oscillatory shear stress tests were conducted at a fixed angular frequency of 10 rad/s to record storage modulus (G'), loss modulus (G''), and complex viscosity.

Swelling behavior

The swelling ratio of the hydrogels was determined using the gravimetric method based on a previously established protocol. The initial dry weight (M_0) of each hydrogel was recorded. Hydrogels were then immersed in PBS at room temperature to swell, and their swollen weight (M_1) was measured. The swelling ratio was calculated as: Swelling ratio (%) = $M_1/M_0 \times 100\%$.

Degradation behavior

To simulate long-term conditions for organoid culture, hydrogels were incubated in complete medium at 37 °C. At predetermined time points (1, 2, 3, 4, 5, and 6 weeks), samples were retrieved, freeze-dried, and weighed (M_1). The weight retention ratio was calculated as: Weight retention ratio (%) = $M_1/M_0 \times 100\%$.

Drug release

At specified time points (1, 2, 3, 7, 14, 21, and 28 d), samples were freeze-dried and dissolved in methanol to prepare storage solutions. The concentrations of Glu and TD-198946 were quantified using high-performance liquid chromatography (HPLC, LC-10Avp, Japan). A methanol-water mobile phase (60:40, v/v) was used at a flow rate of 1.0 ml/min to analyze drug release profiles.

Cell proliferation assay [cell counting kit-8 (CCK-8)]

On days 1, 3, and 5 of culture, CCK-8 (Beyotime, China) reagent (medium:CCK-8 ratio of 1:1) was added to each well and incubated for 2 h at 37 °C. The absorbance of the supernatant was then measured at 450 nm using a multifunctional microplate reader (Biotech CTYTATION5, USA) to quantitatively assess the proliferation of BMSCs.

Cytoskeleton staining

After 1 d of culture, the 4 groups of cell-laden spheres were fixed with 4% paraformaldehyde. Cells were then stained with TRITC Phalloidin (Solarbio, China) for 1 h in the dark to label the cytoskeleton.

3D fluorescence images were captured using a confocal laser scanning microscope to observe cell morphology.

Scratch assay

A 200 μl pipette tip was used to create linear scratches on the wells of the plate. After scratching, the cells were cocultured with the hydrogel for 12 h. Subsequently, the cells were stained with the calcein AM/PI double staining kit and incubated for 30 min. Images were captured using an inverted fluorescence microscope, and the migration rate was calculated using ImageJ software.

Transwell assay

A total of 3×10^4 BMSCs were suspended in 200 μl of serum-free α-MEM and seeded into the upper chamber of a Transwell insert. In the lower chamber, 200 μl of RGD-modified DNA-silk fibroin hydrogel (DSR), Glu-containing DSR (DSRG), TD-198946-containing DSR (DSRT), or DNA-silk fibroin hydrogel sustained-release system (DSRGT) hydrogel samples and 500 μl of α-MEM were added. After a 12-hour incubation, cells were stained with 0.03% crystal violet solution (Servicebio, China) for 5 min. Images were captured using an inverted fluorescence microscope, and cell migration was quantified using ImageJ software.

Alcian blue staining

After fixing the cells with 4% paraformaldehyde, they were stained with 1% Alcian blue (Servicebio, China) for 30 min. The stained plate was then photographed using a multifunctional microplate reader to record the staining effect. For quantitative analysis, the cells were washed with 6 mol/L guanidine hydrochloride to elute the dye, and the absorbance of the elution was measured at a wavelength of 630 nm.

Immunofluorescence (IF) staining

Organoid samples were fixed in 4% paraformaldehyde solution, followed by permeabilization with 0.3% Triton X-100 (Beyotime, China) for 10 min and blocking with 10% goat serum for 2 h. The primary antibody (1:1000) was incubated overnight, followed by incubation with the secondary antibody (1:5000) and TRITC Phalloidin for 1 h at room temperature. After staining with 4′,6-diamidino-2-phenylindole (DAPI; Servicebio, China), images were captured using an inverted fluorescence microscope, and fluorescence intensity was quantified using ImageJ software. The

relevant antibodies are listed in **Additional file 1: Table S2.**

Protein extraction and Western blotting analysis

Tissue samples were immediately frozen in liquid nitrogen and ground into a powder. Proteins from tissue or cells were extracted using RIPA lysis buffer (Beyotime, China) containing protease inhibitors (Beyotime, China) and phosphatase inhibitors (Beyotime, China), and protein concentrations were measured using the BCA Protein Assay Kit (Beyotime, China). After separation by SDS-PAGE, the target protein was transferred to a PVDF membrane (Epizyme, China). Non-specific binding was blocked for 15 min using a blocking solution (Epizyme, China), and the membrane was incubated overnight with the primary antibody (1:1000) at 4 °C. Finally, protein signals were detected using an enhanced chemiluminescence detection system (Servicebio, China) with developing solution. Western blotting results were quantified using ImageJ software. The relevant antibodies are listed in **Additional file 1: Table S2.**

1,9-dimethylmethylene blue (DMMB) assay and enzyme-linked immunosorbent assay (ELISA)

Col II content was measured using a rabbit-derived Col II kit (Cloud-Clone, China), with absorbance measured at 450 nm. For GAG content, 10 μl of the digested sample was mixed with 100 μl of DMMB (Sigma, USA) and incubated at 37 °C for 30 min. The optical density (OD) was then measured at 525 nm to determine the GAG content.

RNA extraction and quantitative real-time polymerase chain reaction (qRT-PCR)

Total mRNA was extracted using Trizol reagent (Beyotime, China), chloroform (Aladdin, China), isopropanol (Aladdin, China), and 75% ethanol (Aladdin, China). Complementary DNA (cDNA) was synthesized through reverse transcription using TaKaRa Reverse Transcription Reagent (TaKaRa, Japan). qRT-PCR was performed using the QuantStudio 6 Flex RT-qPCR system combined with SYBR Green PCR Mix (TaKaRa, Japan). The primer sequences for the relevant genes are provided in **Additional file 1: Table S3**.

Transcriptomics and data processing

Cell samples were directly extracted using Trizol reagent (Beyotime, China). Tissue samples were frozen in liquid nitrogen immediately after collection, ground into powder, and then extracted using Trizol reagent (Beyotime, China). The corresponding RNA libraries were constructed by OE Biotech

Co., Ltd., China, and transcript products were analyzed through high-throughput sequencing. Sequencing data were processed using DESeq software to identify differentially expressed genes (DEGs), with significant genes selected based on a P-value < 0.05 and a $|\log_2$ fold change$| > 1$. Furthermore, principal component analysis, enrichment analysis of Kyoto Encyclopedia of Genes and Genomes (KEGG) pathways, Gene Ontology (GO), and short time-series expression miner (STEM) were conducted employing advanced algorithms within the R package.

Atomic force microscopy (AFM)

All rat femur samples were fixed in 4% formalin for 48 h and then analyzed using the Bruker Dimension Icon system for AFM.

Hematoxylin and eosin (HE) staining, Safranin O/Fast Green staining, and immunohistochemistry (IHC)

Tissue samples were fixed in 4% formalin for 48 h. Rat femur samples were then decalcified for 6 weeks after fixation in 4% formalin. All samples were dehydrated through an ethanol gradient and embedded in paraffin. After embedding, the samples were sectioned into 10 μm-thick slices. For HE staining, samples were stained with HE (Servicebio, China). For Safranin O/Fast Green staining, samples were stained with Safranin O/Fast Green staining(Servicebio, China). For IHC, samples were blocked with 10% goat serum for 2 h, incubated with primary and secondary antibodies, and images were captured using a high-throughput slide scanner (Olympus, Japan). The relevant antibodies are listed in **Additional file 1: Table S2.**

Table S1 DNA sequence information (the bold sequences are the sticky ends)

ssDNA	Sequence (5' – 3')	Length (nt)
**Y1**	CTTACGACGCACAAGGAGATCATGAG**TAACTGGACACTT**	39
**Y2**	CTTACGACGCACAAGGAGATCATGAG**TAACTGGACACTT**	39
**Y3**	CTCATGATCTCCTTTAGGCAGACAGG**TAACTGGACACTT**	39
**L1**	CTACGGTGAATGGAATTCTCATGCGAATAGA**AAGTGTCCAGTTA**	44
**L2**	TCTATTCGCATGAGAATTCCATTCACCGTAG**AAGTGTCCAGTTA**	44

ssDNA single-stranded DNA, *Y1–Y3* ssDNA1, ssDNA2, and ssDNA3, which together constitute the Y-scaffold, *L1–L2* ssDNA1 and ssDNA2, which together constitute the L-linker

Table S2 Antibodies for Western blotting, IF, and IHC

**Name**	**Manufacture**	**Cat No.**	**Application**
Col II	Servicebio	GB11021	Western blotting, IF, IHC
Col X	Abcam	ab49945	IF
Col I	Proteintech	14695-1-AP	IF
ACAN	Proteintech	68350-1-Ig	Western blotting, IF
SOX9	Abcam	ab185966	Western blotting, IF
p-p38 MAPK	Proteintech	28796-1-AP	Western blotting
p38 MAPK	Proteintech	14064-1-AP	Western blotting
p-ERK1/2	Proteintech	80031-1-RR	Western blotting
ERK1/2	Proteintech	11257-1-AP	Western blotting
p-JNK	Proteintech	80024-1-RR	Western blotting
JNK	Proteintech	51153-1-AP	Western blotting
Notch3	Cell Signaling Technology	5276T	Western blotting
GAPDH	Servicebio	GB11002	Western blotting

Col II type II collagen, *COL X* type X collagen, *Col I* type I collagen, *ACAN* aggrecan, *SOX9* SRY-box transcription factor 9, *p-p38 MAPK* phosphorylated mitogen-activated protein kinase p38, *p38 MAPK* mitogen-activated protein kinase p38, *p-ERK1/2* phosphorylated extracellular signal-regulated kinase 1/2, *ERK1/2* extracellular signal-regulated kinase 1/2, *p-JNK* phosphorylated c-Jun N-terminal kinase, *JNK* c-Jun N-terminal kinase, *Notch3* Notch receptor 3, *GAPDH* glyceraldehyde-3-phosphate dehydrogenase, *IF* immunofluorescence, *IHC* immunohistochemistry

Table S3 Primer sequences of the genes for qRT-PCR analysis

Gene	Forward primer (5' – 3')	Reverse primer (5' – 3')
GAPDH	GAAGAAGGTGGTGAAGCAGGG	CACTGTTGAAGTCGCAGGAG
Col II	CACGCTCAAGTCCCTCAACAG	TCTATCCAGTAGTCACCGCTCT
ACAN	GGAGGAGCAGGAGTTTGTCAAA	TGTCCATCCGACCAGCGAAAAC
SOX9	GCGGAGGAAGTCGGTGAAGAATC	AAGATGGCGTTGGGCGAGAT
Col X	AAGTGGACCGAAAGGAGACA	TGGAAACCCATTCTCACCTC
Col I	TGGCAAGAACGGAGATGACG	GCACCATCCAAACCACTGAA

qRT-PCR quantitative real-time polymerase chain reaction, *GAPDH* glyceraldehyde-3-phosphate dehydrogenase, *Col II* type II collagen, *ACAN* aggrecan, *SOX9* SRY-box transcription factor 9, *COL X* type X collagen, *Col I* type I collagen

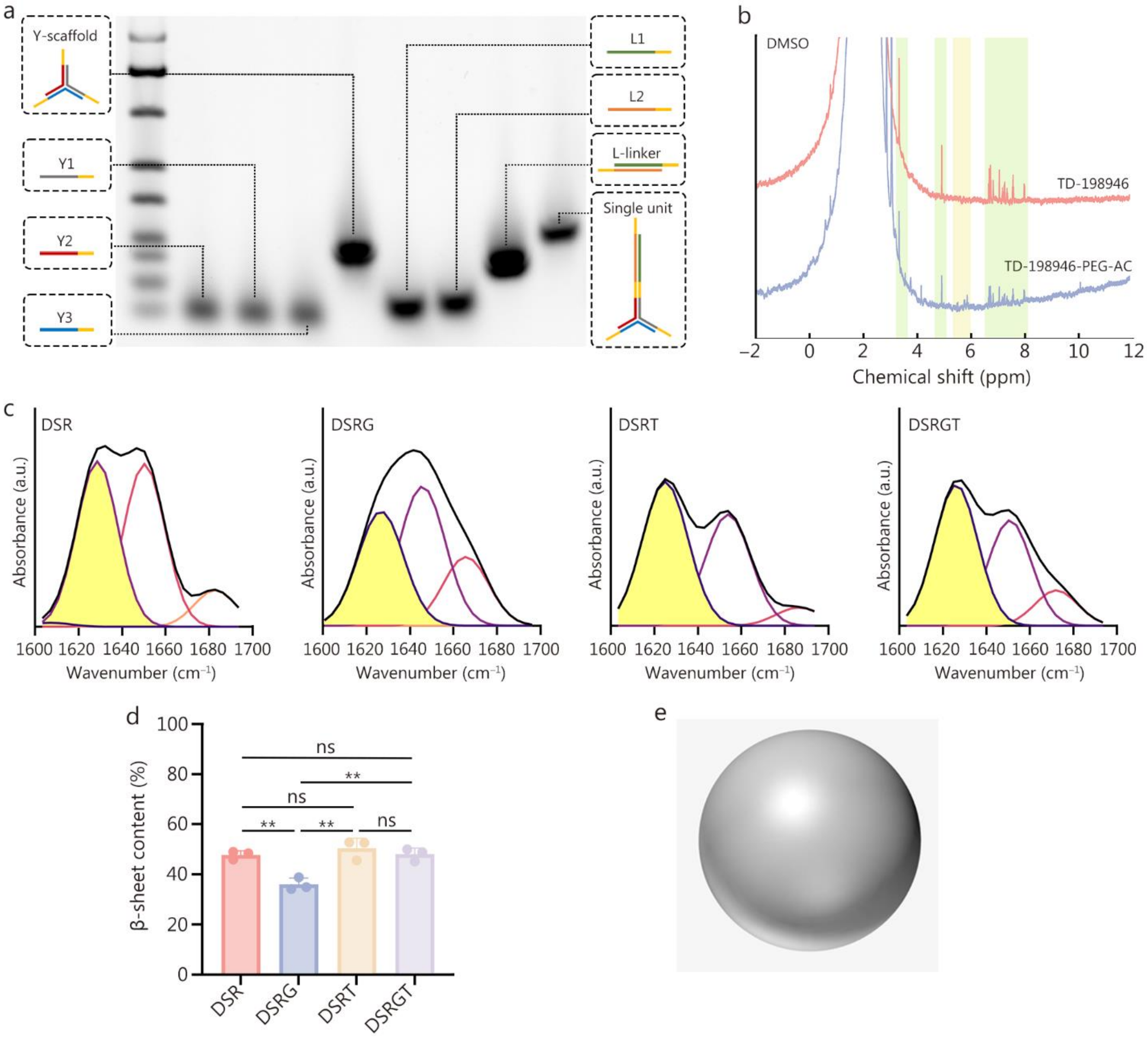

Fig. S1 Synthesis and characterization of DSRGT. **a** Agarose gel electrophoresis characterization of DNA strands. **b** ^{1}H nuclear magnetic resonance (NMR) spectra of TD-198946 (red) and TD-198946-PEG-AC (blue) in DMSO. The green box highlights the characteristic peaks of TD-198946, and the yellow box highlights the characteristic peaks of AC-PEG-NHS. **c** Absorbance spectra of DSR, DSRG, DSRT, and DSRGT deduced after fourier self-deconvolution. The β-sheet component is highlighted by the yellow peaks. **d** β-sheet content of DSR, DSRG, DSRT, and DSRGT ($n = 3$). **e** Overview of 3D-printed cartilage organoids. Data are presented as mean ± SD. DMSO dimethyl sulfoxide, DSR RGD-modified DNA-silk fibroin hydrogel, DSRG Glu-containing DSR, DSRT TD-198946-containing DSR, DSRGT DNA-silk fibroin hydrogel sustained-release system, Glu glucosamine, 3D three-dimensional, AC-PEG-NHS acid-polyethylene glycol-N-hydroxysuccinimide

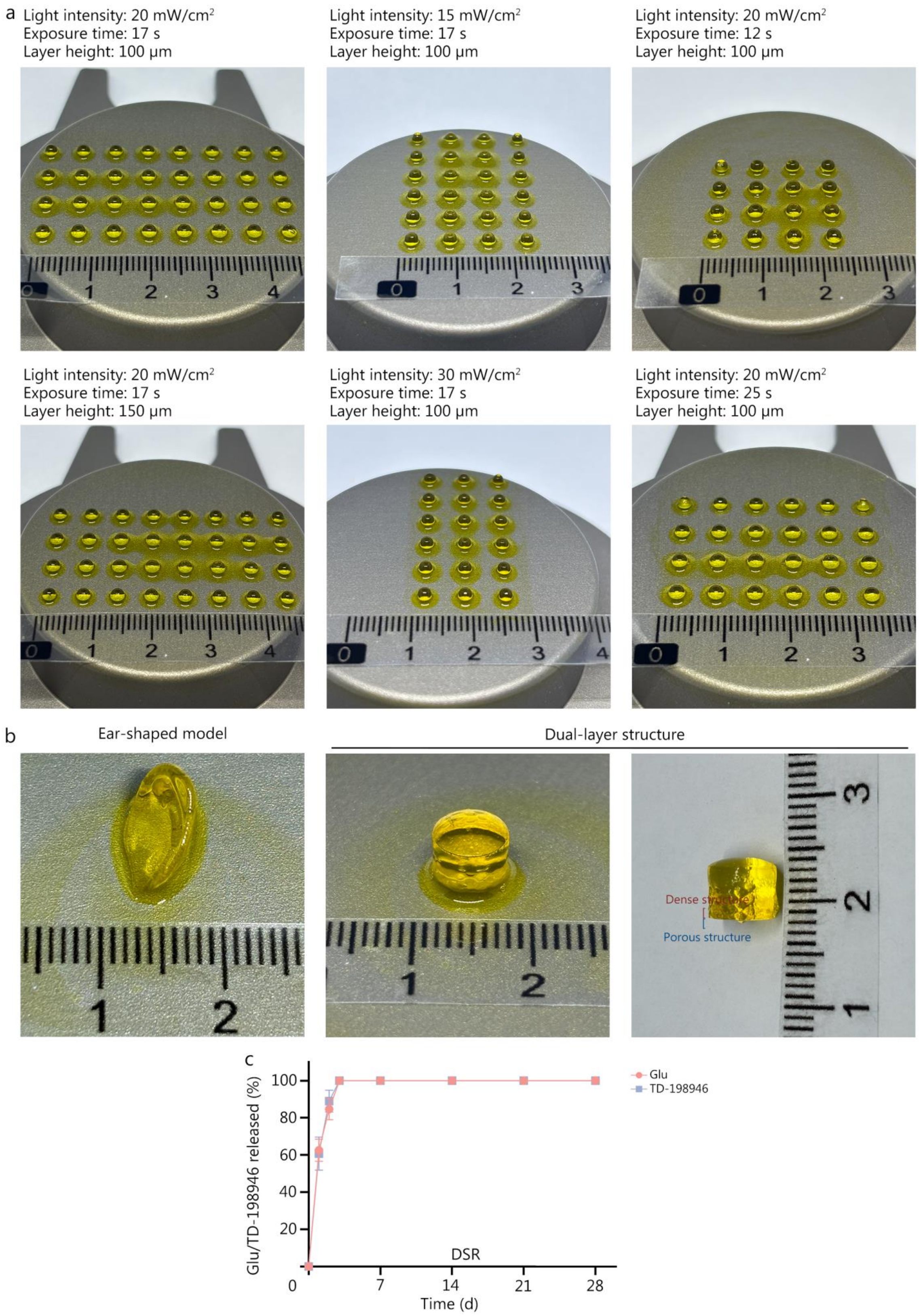

Fig. S2 Models 3D-printed using a DLP system based on DSRGT. **a** 3D printed spherical array under different parameters. **b** Representative images of the 3D printed models. **c** Release profiles of Glu and TD-198946 from DSR (*n* = 6). DLP digital light processing, DSRGT DNA-silk fibroin hydrogel sustained-release system, 3D three-dimensional, DSR RGD-modified DNA-silk fibroin hydrogel, Glu glucosamine

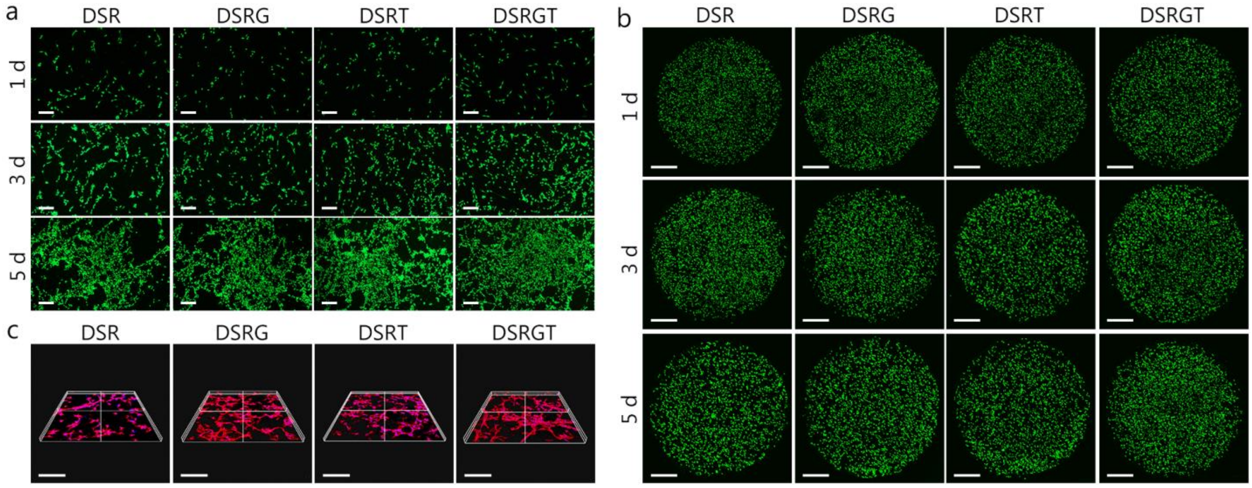

Fig. S3 Effects of DSR, DSRG, DSRT, and DSRGT on BMSC biocompatibility and spreading. **a** Live/dead staining images of BMSCs cultured on DSR, DSRG, DSRT, and DSRGT hydrogels. Scale bar = 500 μm. **b** Live/dead staining images of cell-laden spheres printed using DLP based on DSR, DSRG, DSRT, and DSRGT hydrogels. Scale bar = 500 μm **c** 3D projection images showing the spreading of BMSCs within DSR, DSRG, DSRT, and DSRGT. Scale bar = 100 μm. DSR RGD-modified DNA-silk fibroin hydrogel, DSRG Glu-containing DSR, DSRT TD-198946-containing DSR, DSRGT DNA-silk fibroin hydrogel sustained-release system, BMSCs bone-marrow mesenchymal stem cells, DLP digital light processing, 3D three-dimensional

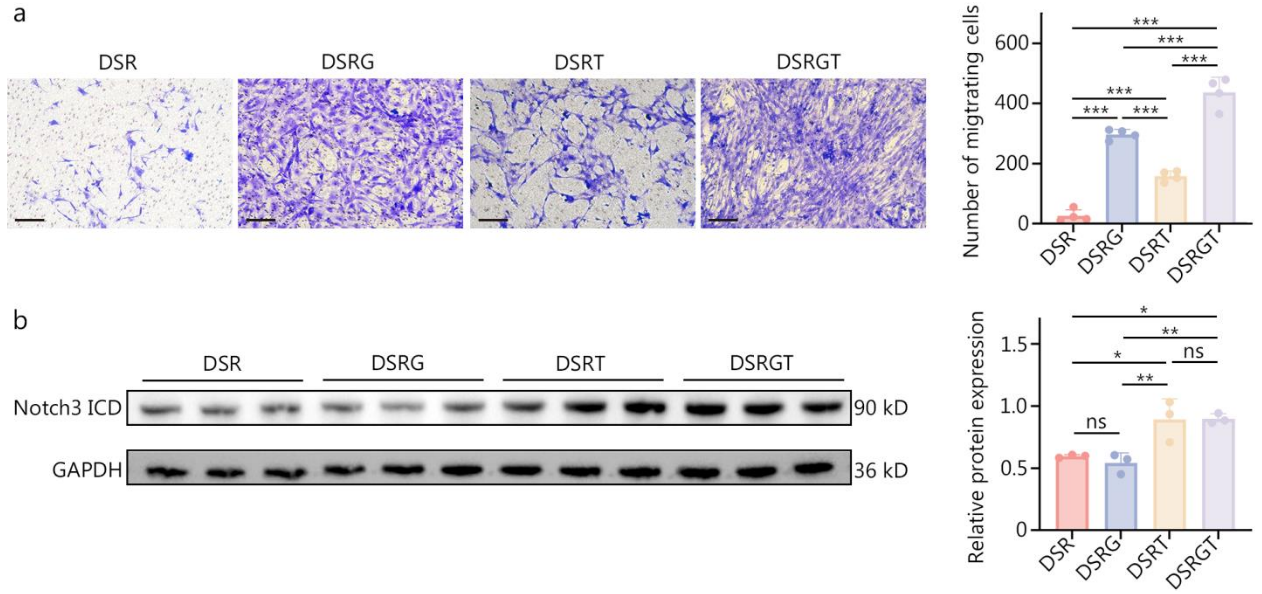

Fig. S4 TD-198946 activates the Notch3 signaling pathway in BMSCs. **a** Transwell assay and quantitative analysis of DSR, DSRG, DSRT, and DSRGT groups (n = 4). Scale bar = 500 μm. **b** Western blotting and quantitative analysis for DSR, DSRG, DSRT, and DSRGT groups (n = 3). One-way ANOVA and Tukey's multiple-comparisons test were used for data analysis. $^{*}P < 0.05$, $^{**}P < 0.01$, $^{***}P < 0.001$, ns non-significant. DSR RGD-modified DNA-silk fibroin hydrogel, DSRG Glu-containing DSR, DSRT TD-198946-containing DSR, DSRGT DNA-silk fibroin hydrogel sustained-release system, BMSCs bone-marrow mesenchymal stem cells, Notch3 Notch receptor 3, Notch3 ICD Notch receptor 3 intracellular domain

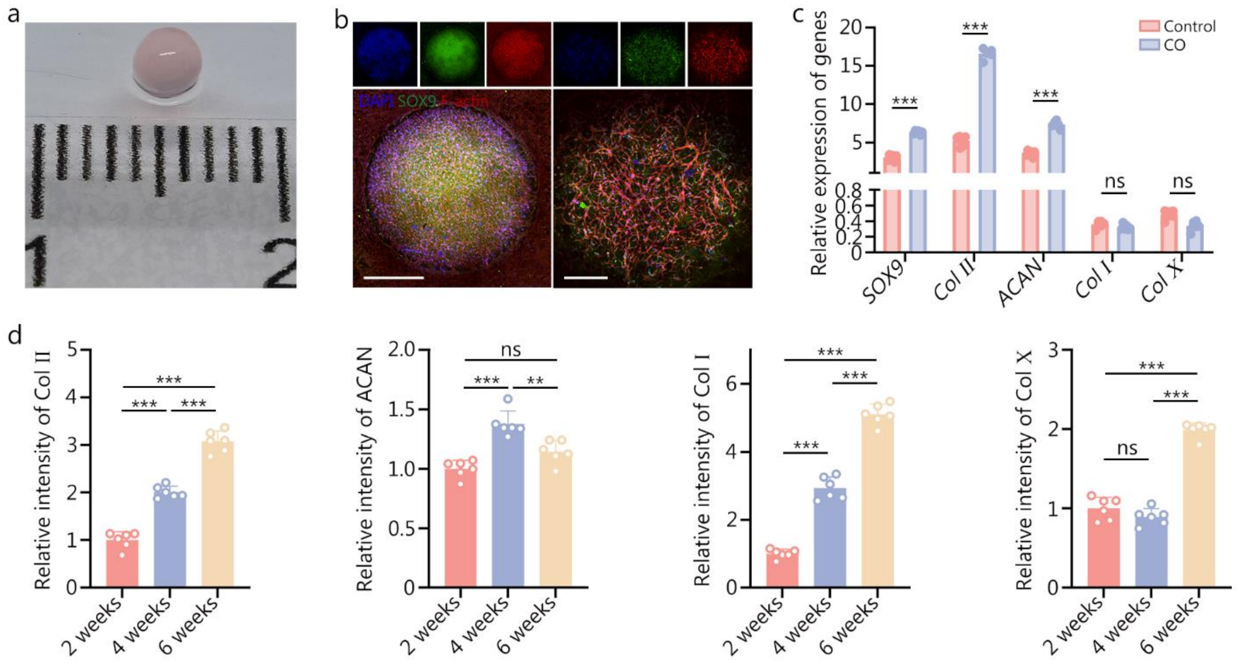

Fig. S5 Evaluation of COs. **a** Macroscopic images of COs cultured for 1 week. **b** Immunofluorescence (IF) staining of SOX9 in COs after 1 week of culture. Scale bar = 1 mm (left) and 200 μm (right). **c** qRT-PCR results for *SOX9*, *Col II*, *ACAN*, *Col I*, and *Col X* expression after 1 week of culture ($n = 6$). **d** Quantitative analysis of IF results for Col II, ACAN, Col I, and Col X of COs cultured for 2, 4, and 6 weeks. Data are presented as mean ± SD. One-way ANOVA and Tukey's multiple-comparisons test were used for data analysis. $^{**}P < 0.01$, $^{***}P < 0.001$, ns non-significant. COs cartilage organoids, Col II type II collagen, ACAN aggrecan, SOX9 SRY-box transcription factor 9, Col X type X collagen, Col I type I collagen

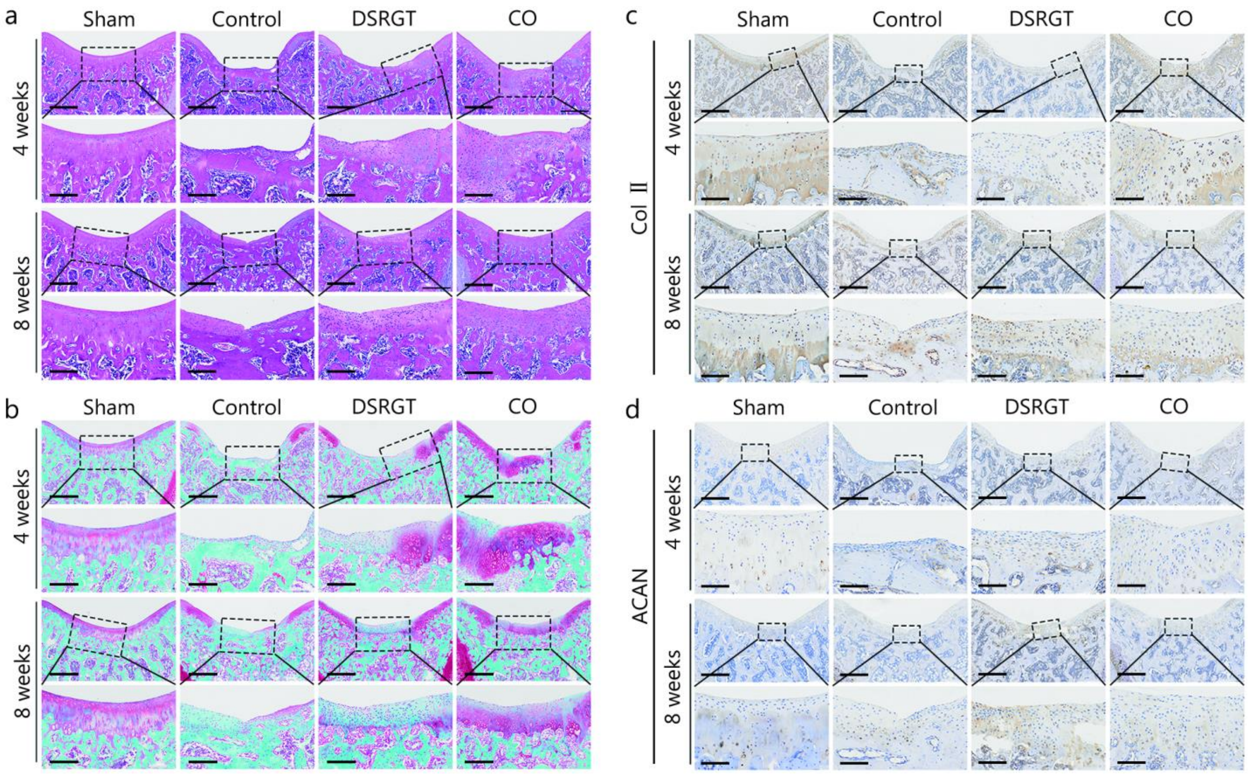

Fig. S6 Histological staining and immunohistochemical (IHC) staining results of regenerated cartilage at 4 and 8 weeks post-treatment. **a** Hematoxylin and eosin (HE) stained image of regenerated cartilage at 4 and 8 weeks post-treatment. **b** Safranin O/Fast Green staining images of regenerated cartilage at 4 weeks and 8 weeks post-treatment. **c** IHC staining for Col II in regenerated cartilage at 4 and 8 weeks post-treatment. **d** IHC staining for ACAN in regenerated cartilage at 4 and 8 weeks post-treatment. Scale bar = 500 μm (upper) and 200 μm (lower). Sham positive control, Control untreated defects covered with fibrin glue, DSRGT defects treated with DSRGT and fibrin glue, CO defects treated with 4-week cultured cartilage organoids and fibrin glue, Col II type II collagen, ACAN aggrecan

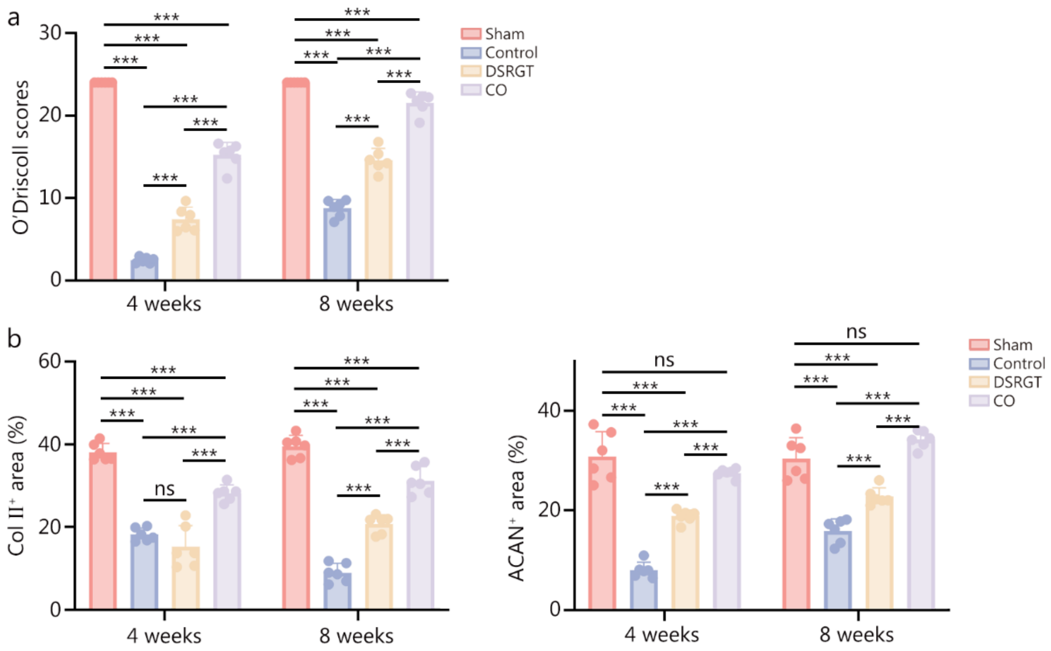

Fig. S7 Evaluation of regenerated cartilage at 4 and 8 weeks post-treatment. **a** O'Driscoll scoring results. **b** Quantitative analysis of immunohistochemical results ($n = 6$). One-way ANOVA and Tukey's multiple-comparisons test were used for data analysis. $^{**}P < 0.01$, $^{***}P < 0.001$, ns non-significant. Sham positive control, Control untreated defects covered with fibrin glue, DSRGT defects treated with DSRGT and fibrin glue, CO defects treated with 4-week cultured cartilage organoids and fibrin glue, Col II type II collagen, ACAN aggrecan

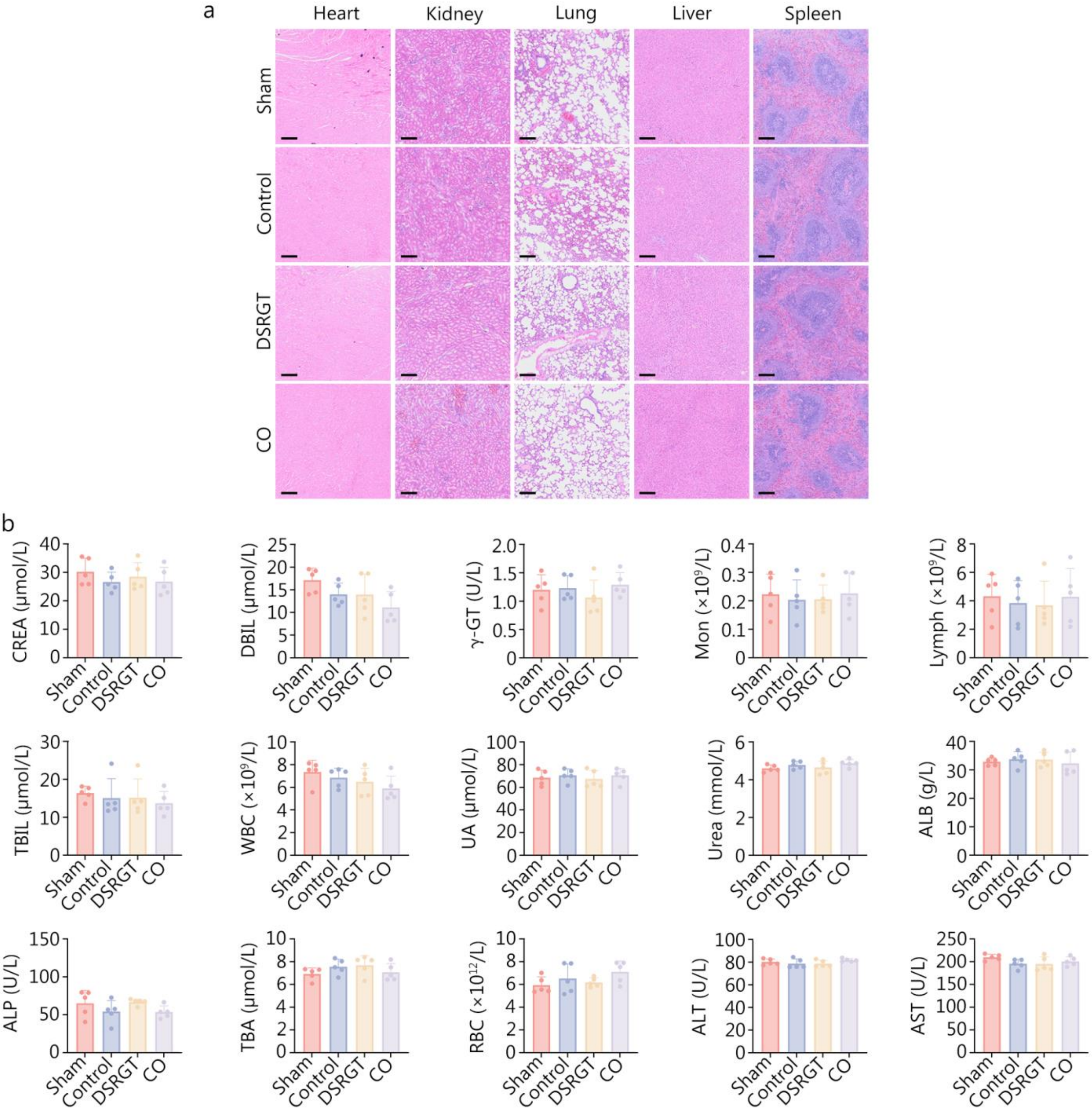

Fig. S8 In vivo toxicity evaluation. **a** Hematoxylin and eosin (HE) stained images of the heart, kidneys, lungs, liver, and spleen in each group at 8 weeks post-surgery. Scale bar = 200 μm. **b** Hematological and liver/kidney function assessments in each group at 8 weeks post-surgery. Sham positive control, Control untreated defects covered with fibrin glue, DSRGT, defects treated with DSRGT and fibrin glue, CO defects treated with 4-week cultured cartilage organoids and fibrin glue, CREA creatinine, DBIL direct bilirubin, γ-GT gamma-glutamyl transferase, Mon monocytes, Lymph lymphocytes, TBIL total bilirubin, WBC white blood cell count, UA uric acid, ALB albumin, ALP alkaline phosphatase, TBA total bile acid, RBC red blood cell count, ALT alanine aminotransferase, AST aspartate aminotransferase

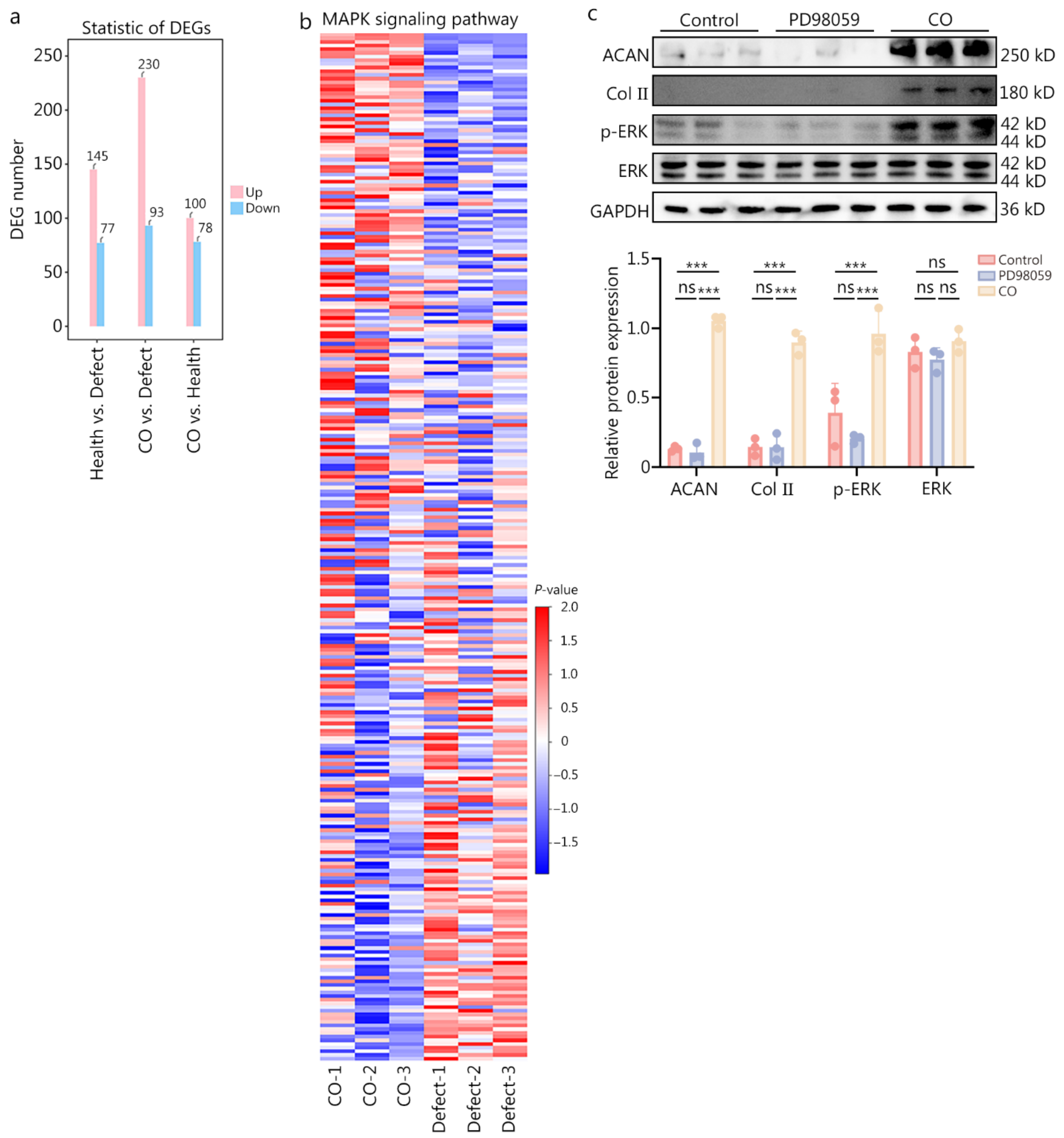

Fig. S9 Cartilage organoids (COs) promote cartilage regeneration by upregulating the MAPK pathway. **a** Statistical summary of DEGs. **b** Clustered gene set enrichment analysis (GSEA) results. **c** Western blotting and quantitative analysis for control, PD98059, and CO groups ($n = 3$). One-way ANOVA and Tukey's multiple-comparisons test were used for data analysis. $^{***}P < 0.001$, ns non-significant. Control uninduced BMSCs, PD98059 cartilage organoids cultured with PD98059 for 4 weeks, CO cartilage organoids cultured for 4 weeks, Col II type II collagen, ACAN aggrecan, GAPDH glyceraldehyde-3-phosphate dehydrogenase, p-ERK phosphorylated extracellular signal-regulated kinase, ERK extracellular signal-regulated kinase 1/2, DEGs differentially expressed genes
